# PAK1 overexpression promotes myxofibrosarcoma angiogenesis through STAT5B-mediated *CSF2* transactivation: clinical and therapeutic relevance of amplification and nuclear entry

**DOI:** 10.7150/ijbs.83467

**Published:** 2023-07-31

**Authors:** Chien-Feng Li, Ti-Chun Chan, Fu-Min Fang, Shih-Chen Yu, Hsuan-Ying Huang

**Affiliations:** 1 Department of Medical Research, Chi-Mei Medical Center, Tainan, Taiwan.; 2 National Institute of Cancer Research, National Health Research Institutes, Tainan, Taiwan.; 3 Institute of Precision Medicine, National Sun Yat-sen University, Kaohsiung, Taiwan.; 4 Department of Radiation Oncology, Kaohsiung Chang Gung Memorial Hospital and Chang Gung University College of Medicine, Kaohsiung, Taiwan.; 5 Department of Anatomic Pathology, Kaohsiung Chang Gung Memorial Hospital and Chang Gung University College of Medicine, Kaohsiung, Taiwan.

**Keywords:** myxofibrosarcoma, PAK1, nuclear entry, STAT5B, CSF2, angiogenesis

## Abstract

Myxofibrosarcoma is genetically complex without established nonsurgical therapies. In public datasets, *PAK1* was recurrently gained with mRNA upregulation. Using myxofibrosarcoma cells, we explored the oncogenic underpinning of PAK1 with genetic manipulation and a pan-PAK inhibitor (PF3758309). Myxofibrosarcoma specimens were analyzed for the levels of PAK1, phospho-PAK^T423^, CSF2 and microvascular density (MVD) and those of *PAK1* gene and mRNA. PAK1-expressing xenografts were assessed for the effects of PF3758309 and *CSF2* silencing. Besides pro-proliferative and pro-migrator/pro-invasive attributes, PAK1 strongly enhanced angiogenesis in vitro, which, not phenocopied by PAK2-4, was identified as CSF2-mediated using antibody arrays. PAK1 underwent phosphorylation at tyrosines^153,201,285^ and threonine^423^ to facilitate nuclear entry, whereby nuclear PAK1 bound STAT5B to co-transactivate the *CSF2* promoter, increasing CSF2 secretion needed for angiogenesis. Angiogenesis driven by PAK1-upregulated CSF2 was negated by *CSF2* silencing, anti-CSF2, and PF3758309. Clinically, overexpressed whole-cell phospho-PAK^T423^, related to *PAK1* amplification, was associated with increased grades, stages, and *PAK1* mRNA, higher MVD, and CSF2 overexpression. Overexpressed whole-cell phospho-PAK^T423^ and CSF2 independently portended shorter metastasis-free survival and disease-specific survival, respectively. In vivo, both CSF2 silencing and PF3758309 suppressed PAK1-driven tumor proliferation and angiogenesis. Conclusively, the nuclear entry of overexpressed/activated PAK1 endows myxofibrosarcomas with pro-angiogenic function, highlighting the vulnerable PAK1/STAT5B/CSF2 regulatory axis.

## Introduction

Myxofibrosarcoma, a sarcoma commonly occurring in the limbs and torso of elderly adults, features multinodular proliferation of fibroblastic cells with variable pleomorphism, mitoses and myxoid stroma rich in curvilinear vessels 1. Without disease-defining hallmarks, myxofibrosarcoma exhibits increased grades and metastases after relentless recurrences with a 5-year overall survival rate of 65-75% 1-3. The aggressiveness of myxofibrosarcoma is coupled with the genomic complexity, particularly the copy number alterations (CNAs), and its wide histological spectrum renders it suitable to model the effects of accumulative aberrations on multistep sarcomagenesis 1, 4-8. The therapeutic mainstay of myxofibrosarcoma is curative resection, currently lacking established chemotherapeutic or targeted therapies 1, 3, 9, 10. Therefore, the discovery of deregulated molecular targets resulting from critical CANs is desirable to introduce novel prognostic adjuncts and therapeutic strategies.

Recently, we integrated the multi-layered evidence to establish a novel oncogenic attribute of overexpressed *RSF1*, which co-opts CEBP/β and hSNF2H to transactivate IL-1β, in turns promoting angiogenesis in myxofibrosarcomas 11. *RSF1* gene is amplified in a significant subset of myxofibrosarcomas and resides on 11q13-14.1, where *RSF1* and *PAK1* are the only two significantly upregulated genes with gained copies in this region 11. However, the clinical and functional relevance of PAK1 and associated signaling remains undefined in myxofibrosarcoma. Compared with RSF1, PAK1, the prototypical member of p21-activating kinase family, is relatively feasible as a direct therapeutic target, since PAK1 functions as a cytosolic molecular hub converging upstream signals mediated by RhoGTPases to integrate various mitogenic and morphogenetic inputs and transcriptionally co-regulate expression of target genes upon nuclear relocation 12-19.

In primary myxofibrosarcoma samples, we clinically validated the whole-cell and nuclear expression of total PAK1 and its phosphorylated form (p-PAK1^T423^), which exhibited significant associations with *PAK1* gene amplification, increases in mRNA abundance, grades and stages, and adverse outcomes. In cell and xenograft models, we corroborated the classical pro-proliferative and pro-migratory/invasive oncogenic attributes of PAK1 and unraveled a novel pro-angiogenic PAK1/STAT5B/CSF2 axis, driven by nuclear expression of activated PAK1 that requires specific tyrosine phosphorylation and undergoes threonine phosphorylation. Upon nuclear entry together with STAT5B, PAK1 co-transactivated the pro-angiogenic *CSF2* gene, encoding granulocyte-macrophage colony stimulating factor 2 (a.k.a GM-CSF), to increase CSF2 expression, hence enhancing angiogenesis in myxofibrosarcomas. This signaling axis also offers a vulnerability targetable by small-molecule PAK inhibitors, such as ATP-competitive PF-3758309 14, 15, which enables inhibition on growth and angiogenesis of myxofibrosarcoma through suppressing expression and phosphorylation of PAK1 to downregulate *CSF2* transactivation.

## Materials and methods

### Reappraisal of published transcriptomic and genomic profiling datasets

Nexus software (BioDiscovery) was employed to reappraise our previously reported genomic profiling data (GSE35483) of myxofibrosarcoma tissue and cell line samples for profiling the DNA copy number alterations across the whole genome and identifying altered genes within imbalanced chromosomes in a zoomed-in view 6, 11. Apart from previously reported 5p gain, an additional gained region was mapped to chromosome 11q, specifically 11q13.5-14.1 region, where several oncogenes were previously described to be amplified in common carcinomas 20, 21. Hence, candidates in the published transcriptomic datasets (GSE21122) were further screened for those exhibiting differential mRNA expression between myxofibrosarcomas and non-neoplastic soft tissues 5, given that genes with concordant alterations in the genomic and expression profiling represent potential drivers leading to myxofibrosarcoma pathogenesis. The raw CEL files obtained from Affymetrix U133A microarray platform were then imported into Nexus Expression 3 software (BioDiscovery) to analyze all probe sets without pre-selection or filtering. Those genes with p<0.001 were considered significant.

### Tumor characteristics

The institutional review boards of Chang Gung (97-1110A3) hospital granted research use of tumor tissues under anonymous processing. Without neoadjuvant therapy, primary myxofibrosarcomas were resected with curative intent in 114 patients whose formalin-fixed tumor specimens were assembled into tissue microarrays (TMA). TMA sections were employed for *PAK1*-specific fluorescence in situ hybridization (FISH) and the immunohistochemical assessment of the expression levels and subcellular localization of PAK1, phosphorylated PAK1 at residue 423 (p-PAK1^T423^), and CSF2, and CD31-highlighted microvessel density (MVD).

### FISH

FISH assay was performed using a bacterial artificial chromosome-based red probe (CTD-2340N2, Invitrogen) targeting *PAK1* at 11p13.5-14.1. A reference green probe (CTB-28I9, Invitrogen) targeting a region near ZNF725 in 19p12 was used, given no CNAs in prior genomic profiling. In a given specimen, amplification was defined as the ratio of red signals to green signals exceeding 5 by examining 200 tumor cells.

### Immunohistochemistry

TMA sections were microwave-heated, incubated with primary antibodies against total PAK1 (Cell signaling, 2602, 1:200), phospho-PAK1 against Threonine at residue 423 (p-PAK1^T423^, Cell signaling, 2601, 1:200), CSF2 (Abcam, ab300495, 1:100), and CD31 (BD Pharmingen, 550274, 1:100), and detected for protein immunoexpression using the Dako EnVision kit. In each case, we assessed the mean percentages of labeled cells in the tumoral cytoplasm and/or nuclei in triplicate TMA cores, referred to as the labeling index (LI) of tumor cells detected by individual antibodies. Overexpression of whole-cell PAK1 or whole-cell p-PAK1^T423^ was defined when the sum of cytoplasmic LI and nuclear LI with moderate or strong intensity was ≧50%. Specifically for nuclear p-PAK1^T423^, nuclear LI ≧10% was considered positive. CSF2 overexpression was called when moderate or strong cytoplasmic LI was observed in >50% of tumor cells. We conducted computerized analysis to quantify tumoral microvessel density (MVD) of the CD31-stained vessels by ImageJ software, which calculated the mean area (μm^2^) per vessel after normalization to a 0.20 m^2^ field 22.

In formalin-fixed xenografted specimens, whole sections from were stained with anti-Ki67 (Cat. No. 14-5698-82, Clone SolA15, 1:100, Invitrogen), while total PAK1, p-PAK1^T423^, CSF2, and anti-CD31 were stained and scored following the methods used in TMA sections.

### Quantigene Branched-chain DNA in situ hybridization (bDISH) assay

As detailed in **Supplementary Method-S1**, this nucleic acid hybridization technology was utilized to quantitate the mRNA abundance of *PAK1* and housekeeping genes in tissue homogenates of formalin-fixed specimens 23. Specific probes targeting the *PAK1* transcript were customized by QuantiGene Multiplex 2.0 assay system (Affymetrix/Panomics). The detected readout of *PAK1* mRNA abundance was further normalized by that of reference *GAPDH* transcript.

### Cell culture, RNA interference, and transfection

The derivation and maintenance of the OH931, NMFH-1, and NMFH-2 myxofibrosarcoma cell lines, CCD966SK dermal fibroblasts, and human umbilical venous endothelial cells (HUVECs) have been previously reported. Short tandem repeat genotyping was conducted for authentication.

The details of ectopic transfection and stable silencing are provided in **Method-S2.** The pCMV-PAK1 vector or the empty control (Addgene) was stably transfected by Lipofectamine 2000 (Invitrogen) into OH931, NMFH1 or NMFH2 cell line selected with neomycin, while hyperactivated pCMV6-PAK1^T423E^ or pCMV6-PAK1^Y3F^ mutant was created by site-directed mutagenesis for transfection into endogenously PAK1-underexpressing NMFH2 cells.

We stably transduced pLKO.1-*shLacZ* or pLKO.1-*shPAK1* (National RNAi Core Facility, Taiwan) into endogenous PAK1-overexpressing OH931 and NMFH1 cells. These lentiviral vectors were transduced with Lipofectamine 2000 and selected with puromycin. pLKO.1-*shPAK2,* pLKO.1-*shPAK3*, and pLKO.1-*shPAK4* were similarly transduced into OH931 and NMFH1 cells, as was pLKO.1-*shPAK1*. Stable PAK1 transfectants or corresponding controls of NMFH1, NMFH2, and OH931 cell models were transiently transduced with predesigned small interfering RNAs (Ambion) against *STAT5* (*siSTAT5*), *CSF2* (*siCSF2*) or scramble control (*siCtrl*) using Lipofectamine® RNAiMAX reagent. The efficacy of these genetic manipulations was examined by quantitative reverse-transcription polymerase chain reaction (qRT-PCR) and western blots and by Sanger sequencing for mutant vectors.

### qRT-PCR

As detailed in **Method-S3**, we used ABI StepOnePlus™ System to perform real-time RT-PCR for quantitating mRNA abundance of *PAK1-4, STAT5b*, and *CSF2* using customized probes (Thermo Fisher, MA) in myxofibrosarcoma cell line samples with genetic manipulation for PAK1 expression and activity, pharmacological inhibition with PF3758309, or their corresponding controls.

### Human angiogenesis antibody array

Proteome Profiler^TM^ Human Angiogenesis Array Kit (R&D, ARY007) was applied to search for differentially expressed angiogenesis-regulated molecules between *shPAK1* and *shLacZ* transductions for both PAK1-overexpreassing OH931 and NMFH-1 cell lines. According to manufacture's instruction, the angiogenesis antibody membrane, spotted with 55 immobilized angiogenesis-associated antibodies in duplicate, was processed as detailed in **Method-S4.**

### Western blots

To evaluate the effects of genetic manipulations and pharmacologic treatment and the physical interaction of PAK1 with STAT5B in myxofibrosarcoma cell models, equal amounts of proteins extracted from cell lysates were separated by SDS-10% PAGE and transferred to nitrocellulose membranes. Next, the membranes were blocked with skim milk and probed overnight using antibodies against PAK1, p-PAK1^T423^, STAT5B, CSF2, and caspases-3, followed by incubation with the secondary antibody. The dilution of antibodies and blotting procedures are described in **Method-S5**.

### Luciferase reporter assay

The pGL4-phCSF2 promoter construct (Riken, Japan), spanning the critical region between -1383 to +35 relative to the transcription start site, was used to evaluate *CSF2* transactivation in various genetically manipulated cell models. To ensure the specificity of STAT5 binding in transactivating *CSF2*, a pGL4-ph*CSF2* promoter variant lacking the most critical STAT5 binding site (pGL4-ph*CSF2*-Del-193/-179) was created by site-directed mutagenesis. The pGL4 vector, inserts, primers, cotransfection with the reference Renilla vector and analysis of *CSF2* promoter activity are detailed in **Method-S6**.

### Quantitative chromatin immunoprecipitation (qChIP) for *CSF2* promoter

A chromatin immunoprecipitation kit (Millipore) was coupled with qPCR to perform qChIP assays on the precipitated chromatin DNA from various myxofibrosarcoma cells with various manipulations for PAK1 or with PF-3758309 treatment. The ChIP procedures, sources and dilutions of applied antibodies, and primer sequences in qPCR are provided in **Method-S7.**

### Co-immunoprecipitation (co-IP)

Co-IP assays were performed using Dynabeads® Protein G to immunoprecipitate the lysates from OH931, NMFH1, and NMFH2 cell lines, with anti-PAK1 (Cell signaling) coupled to the beads with DMP (Sigma) as cross linkers. The procedures of co-IPs and dilution of antibodies in IPs and western blots are described in** Method-S8.**

### Confocal immunocytochemistry

Confocal immunoflorescent microscopy (FV10i and FV3000, Olympus, Japan) was used to visualize the redistribution of PAK1 expression between subcellular nuclear and cytosolic compartments following pCMV6-PAK1^Y3F^ transfection in NMFH2 cells and to ascertain the co-localization of PAK1 with STAT5 in all three parent myxofibrosarcoma cell lines. The above cells were grown, fixed, incubated with primary and secondary antibodies, counterstained with DAPI, and visualized as described in** Method-S9.**

### In vitro pharmacological assays

PF-3758309 was obtained from Sigma-Aldrich. OH931 and NMFH-1 myxofibrosarcoma cells were seeded onto 96-well plates at a density of 5 x 10^3^ cells/well in complete medium the day before incubation with vehicle control (0.9% saline) or PF3758309 at indicated doses, ranging from 0.01 μM to 10 μM, for 72 h using XTT assay (Roche).

A CSF2-neutralizing antibody (10μg/ml, R&D) or IgG control (mouse IgG, Millipore) was used to incubate HUVEC for 24 h at 37^∘^C with 5% CO_2,_ where the differences in capillary tube-forming capability of HUVEC between anti-CSF2 and IgG treatment were analyzed for various exposures to conditioned media collected from stable pCMV6-PAK1 or empty pCMV6 transfectants of OH931, NMFH1, or NMFH2 cells.

### Functional assays

To elucidate functional alterations associated with PAK1 expression and activation, PAK1 knockdown, CSF2 knockdown, and treatment with PF3758309, various cancer phenotypes were evaluated using bromodeoxyuridine (BrdU), wound-healing, Matrigel invasion, cell cycle kinetic, Annexin V-stained flow cytometric, and HUVEC-based angiogenic assays as appropriate and described previously 6, 11, with modifications detailed in **Methods-S10-S15**.

### Animal xenografts

The protocol was approved by the animal use committee of Chi-Mei Hospital (98121505). To substantiate the oncogenic effects of PAK1 mediated by CSF2 in vivo, PAK1-deficient NMFH2 cells stably pre-transfected with empty vector and transduced with *siCtrl* vs. those with pCMV6-PAK1 and *siCSF2* or *siCtrl* were harvested, resuspended in RPMI-1640, and mixed in a 1:1 PBS and Matrigel mixture, yielding a total volume of 0.2 mL per injection. After anesthesia with isoflurane, 4×10^6^ cells each were separately inoculated into the flanks of eight-week-old male SCID mice (BioLasco, n=8 for each group of different PAK1/CSF2 combinations) and allowed to grow until 19 days after implantation of genetically manipulated NMFH2 cells. To validate the in vivo therapeutic effect of PF3758309**,** 10^6^ NMFH-1 myxofibrosarcoma cells each in 0.1 ml matrigel/PBS were inoculated into the flanks of 30 eight-week-old SCID mice and allowed for growth of xenografted tumors for 7 days, which were then randomized into three groups receiving oral gavage of PF3758309 (0.5 μM or 1 μM) or PBS vehicle control. The treatment was continued until sacrifice on Day 26 post-treatment. The tumor volume was calculated using the formula: V=π/6 x length (mm) x width (mm).

### Statistical Analysis

We evaluated the associations of *PAK1* gene dosage, *PAK1* mRNA abundance, immunoexpression levels of whole-cell PAK1, whole-cell p-PAK1^T423^, nuclear p-PAK1^T423^, cytoplasmic CSF2, and CD31-stained MVD with one another and with clinicopathological factors by using the Chi-square or Wilcoxon rank-sum test as appropriate. Follow-up data were available in 97 cases informative for immunohistochemical data (2-229 months; median, 34.8 months), 68 for *PAK1* gene copies (median, 34.4 months; range, 3.0 to 229), and 61 for *PAK1* mRNA abundance (median, 30.8 months; range, 3.0 to 192). The endpoints were disease-specific survival (DSS) and metastasis-free survival (MeFS). We compared univariate prognostic analyses using the log-rank test. In the multivariate Cox regression analysis, significant prognosticators with univariate p<0.05 were generally included, while tumor size and mitosis, as components of staging and grading, were omitted. In addition, whole-cell p-PAK1^T423^ expression LI was opted as the single parameter representative of PAK1 deregulation in the multivariate Cox regression model, given the robust interdependent covariate relationships among PAK1 aberrations at various levels, fewer cases informative for *PAK1* gene and mRNA alterations, and more global representativeness of whole-cell p-PAK1^T423^ to reflect its pleotropic oncogenic functions that are not wholly attributable to nuclear localization. Student's t-test was used to analyze quantitative RT-PCR, functional, and pharmacological assays for cell and xenograft samples.

## Results

### Elevated copy number and mRNA level of *PAK1* contributes to pro-metastatic, pro-proliferative, and pro-angiogenic phenotypes in myxofibrosarcomas

**Figure 1:**
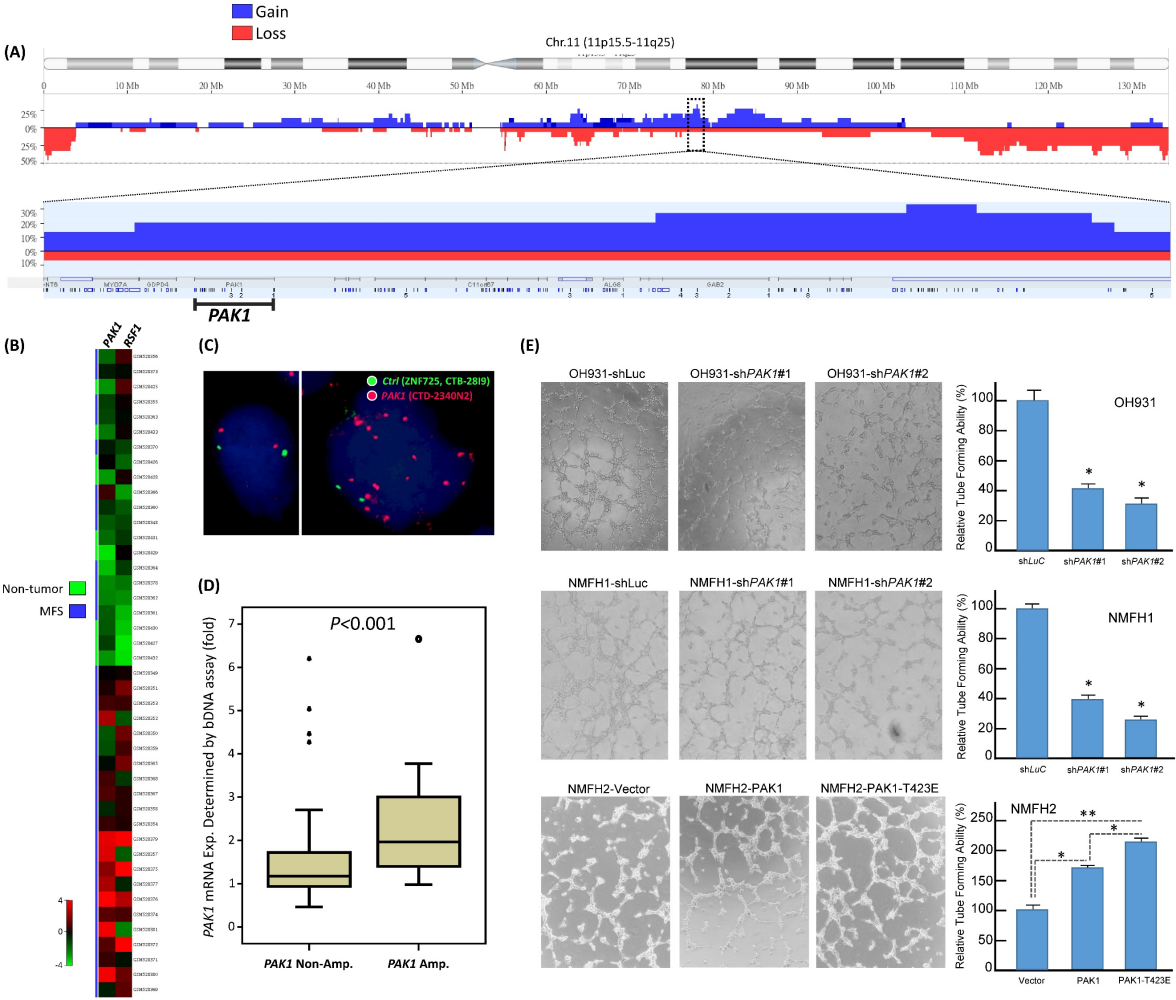
** Public genomic and transcriptomic datasets revealed amplification and differential upregulation of *PAK1* in myxofibrosarcomas with clinical validation in tumor samples and functional evidence of its pro-angiogenic attribute in myxofibrosarcoma cell lines. (A)** Reappraisal of published genomic profiling dataset (GSE35483) unveiled variable but recurrent DNA gains spanning 11q13.5-14.1 amplicon (*dotted rectangle*) in myxofibrosarcoma tumors and cell lines, wherein *PAK1,* alongside *RSF1*, exhibited gained copies in 20% of samples in the zoom-in view. Log2 ratio > 0.2 was used to denote DNA copy gain.** (B)** Regarding genes in the 11q13.5-14.1 amplicon, the heatmap of expressing profiling revealed significantly upregulated expression of *PAK1*, together with *RSF1*, in myxofibrosarcomas, as compared with adjacent normal soft tissues. The filtered criteria were log_2_ fold change ≥0.1 or ≤-0.1 and *P*-value <0.01 **(C)** Fluorescent in situ hybridization demonstrated a normal copy number with 2 paired red/green signals (*left*) and *PAK1* amplification with a signal ratio of red/green probes being >5 (*right*) in one each of grade 1 and grade 3 cases, respectively.** (D)** When normalized to adjacent non-neoplastic tissues, the *PAK1* mRNA expression level, as quantitated by the QuantiGene® bDNA assay, was significantly higher in *PAK1*-amplified myxofibrosarcoma samples than that of *PAK1*-normal cases (*P* <0.001). **(E).** In the HUVEC-based angiogenic assay, the formation of capillary tubes was significantly abrogated when exposed to conditioned media collected from the PAK1-expressing OH931 (*upper*) and NMFH1 (*middle*) cell lines stably transduced with either of both *shPAK1*-bearing lentiviral vectors, as compared with their corresponding *shLacZ*-transduced controls. In contrast, forced expression of pCMV-PAK1 and pCMV-PAK1T^423E^ in NMFH2 cells resulted in prominently increased capillary tubes, in comparison to the empty control, with the degree of angiogenesis achieved by pCMV-PAK1^T423E^ being also significantly higher than that by pCMV-PAK1. The individual histograms summarize the results of triplicate assays for the corresponding experimental conditions of PAK1 knockdown, overexpression and hyperactivation, which were expressed as the mean ± SD. *, *P*<0.05.

From published genomic datasets, copy number gains of myxofibrosarcoma tissues and/or cell samples were recurrently found to span 11q13-14.1 (**Figure-1A**), where several oncogenes recurrently exhibited increased copies, including *PAK1* and previously published *RSF1* 6, 11. We further delved into the concordance between genetic and expression alterations of *PAK1* in two independent cohorts. First, through a focused reappraisal of myxofibrosarcomas relative to non-neoplastic tissues in the published GSE21122 dataset 5, we identified *PAK1*, alongside *RSF* only, as significantly upregulated in the mRNA abundance among candidates on 11q13-14.1 (**Figure-1B**). In our myxofibrosarcoma samples assembled in TMAs, *PAK1* gene copy number and *PAK1* mRNA abundance were assessed using FISH and bDISH assays, respectively, in which *PAK1-*amplified cases (**Figure-1C**), present in 15.5% (11/71) of cases informative for both parameters (**Table-S1**), exhibited a significantly higher *PAK1* mRNA level (**Figurge-1D,** p<0.001), hence indicating *PAK1* as a diver oncogene.

To evaluate the biologic significance of PAK1 and its functional redundancy potentially overlapped with other members of PAK oncogene family, we first characterized three myxofibrosarcoma cell lines (OH931, NMFH1, NMFH2) for their endogenous expression levels of PAK1-4. Compared with reference CCD966SK dermal fibroblasts, OH931 and NMFH1 cell lines exhibited overtly higher mRNA and protein expression levels of *PAK1* (**Supplementary Figure-S1A**), *PAK3,* and *PAK4* (**Supplementary Figure-S2A**) as determined by qRT-PCR and western blot assays, while the expression of endogenous *PAK2* at both mRNA and protein levels was subtly and modestly upregulated in NMFH1 and OH931, respectively. In contrast, NMFH2 showed low levels of endogenous *PAK1* mRNA and protein and consistent deficiency in the protein abundance of PAK2-4, albeit with *PAK3* mRNA upregulation (**Supplementary Figures-S1A, S2A**).

To assess their impact on the essential cancer phenotypes, we successfully transduced two efficient clones each of *shPAK1*-*4* versus *shLacZ* into OH931 and NMFH1 cells (**Figures-S1B, S2B**). In transwell assays, transduction of both *shPAK1* clones consistently and significantly decreased the migratory and invasive capability of OH931 and NMFH1 cells, as compared with* shLacZ* controls (**Figure-S1C, 1D**). Nevertheless, both *shPAK4* clones only attenuated cell migration alone in NMFH1 and OH931 cells but did not consistently inhibit cell invasion, and neither *shPAK2* nor *shPAK3* consistently caused significant abrogation of cell migration or invasion (**Figures-S2C, S2D**). Moreover, both *shPAK1* clones also prominently diminished the BrdU-incorporating rates of NMFH1 and OH931 cells as early as 24 h post-transduction (**Figure-S1E**), while cell proliferation was not consistently abolished in both cell models by *shPAK2-4* and only significantly inhibited at 72 h post-transduction in *shPAK3*- or *shPAK4-*transdued NMFH1 cells (**Figures-S2E**). Angiogenic switch is a critical process in sustained tumor growth and ensuing metastasis 24 and remains less clarified for its implication in the context of PAK1 overexpression/activation in soft tissue sarcomas. As myxofibrosarcomas is a tumor entity featuring prominent vascular network 1, HUVEC assays were applied to analyze the potential anti-angiogenic effects of *shPAK1-4* on the network formation of endothelial tubes under exposure to conditioned media from myxofibrosarcoma cells. In comparison with *shLacZ* controls, only *shPAK1* transduction by either clone could significantly impair the capillary tube-forming capability in both OH931 and NMFH1 models (**Figurge-1E**), while *shPAK2-4* did not influence angiogenesis (**Figures-S3A-C**).

The above knockdown approach validated the consistent oncogenic attributes of overexpressed PAK1 in myxofibrosarcomas cells by conferring pro-proliferative, pro-metastatic, and pro-angiogenic functionality independent of other PAK kinases. To elucidate the biologic impact of overexpressed and hyperactivated PAK1, pCMV6-PAK1, pCMV6-PAK1^T423E^, or empty pCMV6 vector was stably transfected into the endogenously PAK1-underexpressing NMFH2 cells. These genetic manipulations (**Figure-S4A**) were proved successful by qRT-PCR and western blots and by Sanger sequencing for the site-directed mutagenesis to create PAK1^T423E^ mutant, through which pCMV6-PAK1^T423E^ significantly induced greater increases in cell proliferation, migration, and invasion of NMFH2 cells (**Figure-S4B-D**) than pCMV6-PAK1, using the empty pCMV6 as the control. Notably, the pro-angiogenic effect of overexpressed and activated PAK1 was also substantiated in NMFH2 cells, again with a greater amount of HUVEC tubes seen in the transfectant of pCMV6-PAK1^T423E^ than that of pCMV6-PAK1 (**Figure-1E**).

### PAK1 nuclear entry promotes angiogenesis of myxofibrosarcoma by interacting with STAT5B to co-transactivate *CSF2*

**Figure 2:**
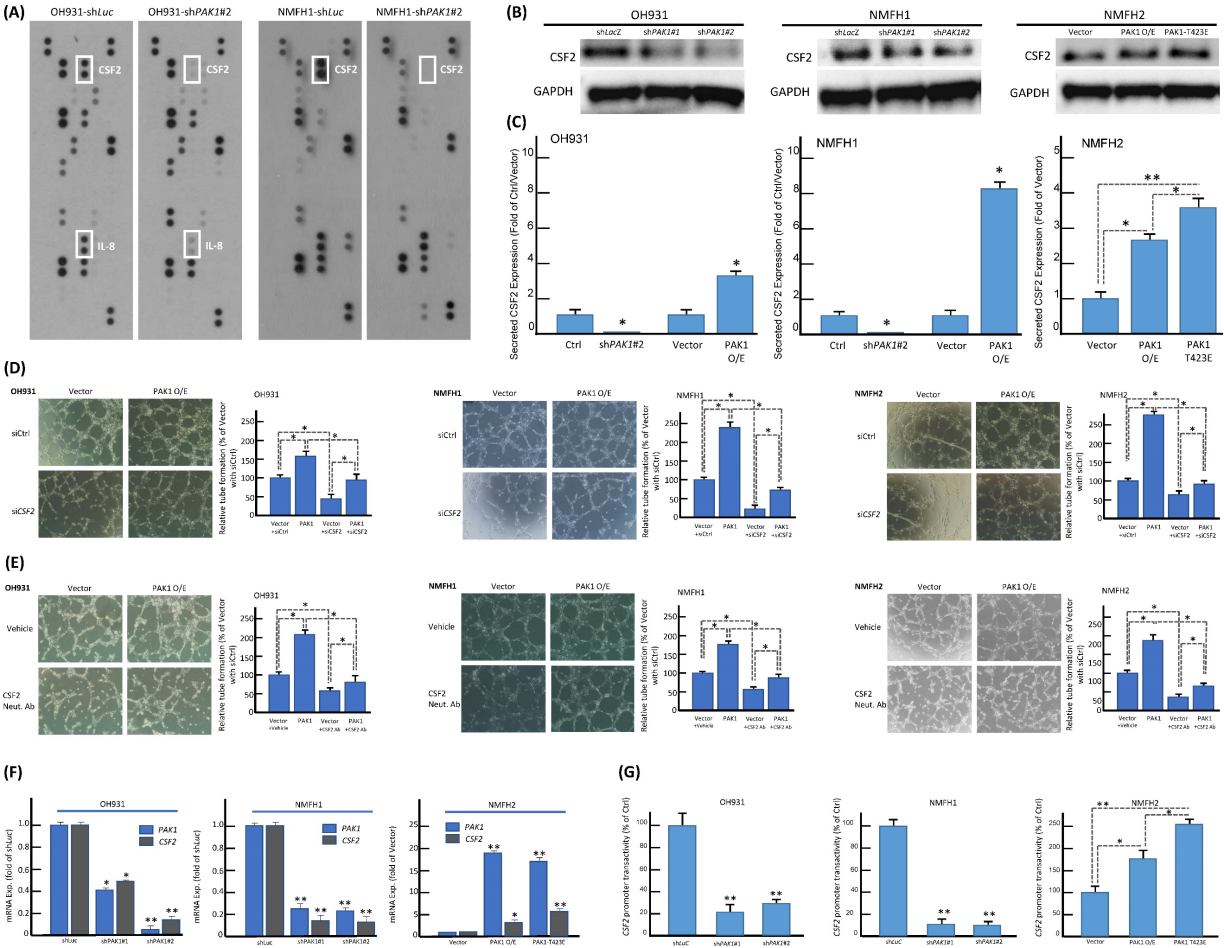
** The identification of overexpression, secretion, and transcriptional upregulation of CSF2 as the downstream mediator of PAK1 in promoting angiogenesis. (A)** In the angiogenesis antibody array, dot blotting analysis illustrated consistent abrogation of CSF2 protein expression in both NMFH1 and OH931 cell models stably transduced with *shPAK1*, compared with the corresponding *shLacZ* controls. However, the IL8 protein expression was only abolished by* shPAK1* in the OH931 cells alone. **(B)** Western blots showed prominently decreased abundance of CSF2 protein in both NMFH1 and OH931 cells stably transduced with either of two *shPAK1* clones, while wild-type pCMV-PAK1 and pCMV-PAK1^T423E^ both resulted in increased CSF2 protein expression in the transfectants of NMFH2 cells.** (C)** Compared with the empty or *shLacZ* controls, the levels of secreted CSF2, as quantitated by ELISA assays, were consistently reduced by *shPAK1* in both NMFH1 and OH931 cells. Similarly, forced expression of wild-type pCMV-PAK1 in all cell lines, as well as pCMV-PAK1^T423E^ transfection in NMFH2 cells, lead to significant increased secretion of CSF2 protein in the corresponding conditioned media. (**D, E**) Targeting CSF2 with genetic ablation or neutralizing anti-CSF2 abrogated the pro-angiogenic capacity contributed by PAK1. In the HUVEC-based angiogenic assays, *siCSF2*-mediated silencing (D, non-targeting *siCtrl* controls) and the anti-CSF2 antibody (E, compared with the vehicle control) similarly caused significant abrogation of capillary tubes formed by both the empty and PAK1 transfectants of NMFH1, OH931, and NMFH2 cells. (**F, G**) As illustrated in the histograms, the *CSF2* mRNA expression level (F) and the *CSF2* promoter activity (G) were both significantly reduced by either of two *shPAK1* clones in OH931 and NMFH1 cell models but elevated in NMFH2 transfectants of wild-type pCMV-PAK1 and pCMV-PAK1^T423E^. Note that pCMV-PAK1^T423E^ induced more prominently increased *CSF2* promoter activity than wild-type pCMV-PAK1 in NMFH2 cells. *, *P*<0.05; **,* P*<0.01.

The consistent in vitro pro-angiogenic attribute of PAK1 seen in genetically manipulated myxofibrosarcoma cell models prompted us to explore the potential angiogenic mediator(s) and pertinent regulatory underpinning. First, we applied a human angiogenesis antibody array to profile the differentially expressed proteins between *shLacZ* and *shPAK1* transductions in NMFH1 and OH931 cells. Among 55 molecules profiled, CSF2, a macrophage differentiation- and angiogenesis-regulating cytokine, represented the sole candidate concurrently downregulated in both cell models upon stable PAK1 knockdown. However, the expression level of IL-8 only decreased in OH931 cells (**Figure-2A**), and the expression levels of VEGF and VEGF-C were either insignificantly altered or generally undetectable in both OH931 and NMFH1 cell models when comparing the conditions of *shPAK1* and *shLacZ* (data not shown)*.* As validated by western blots, both *shPAK1* clones significantly decreased the abundance of total CSF2 protein, while pCMV6-PAK1 and pCMV6-PAK1^T423E^ exhibited overt CSF2-elavating effects (**Figure-2B**). In parallel, secreted CSF2 levels determined by ELISA were singifanlty abolished in *shPAK1*-trasnduced NMFH1 and OH931 cells but significantly raised by pCMV6-PAK1 in all cell models and, to higher extent, in pCMV6-PAK1^T423E^-transfected NMFH2 cells (**Figure-2C**). In HUVEC assays, transient *siCSF2* transduction, in comparison with scramble control, significantly abrogated the capillary tube-forming capacity in all three stable pCMV6-PAK1 transfectants and, to a lesser degree, in empty controls, supporting the angiogenesis-mediating effect of CFS2 (**Figure-2D**). Similarly, the CSF2-neutralizing antibody recapitulated the anti-angiogenic effect of *siCSF2* in all three cell models in the presence or absence of forced PAK1 overexpression (**Figure-2E**) but imposed no significant effects on cell viability and migration/invasion (**Figure-S5A-C**).

Using confocal immunofluorescence microscopy, we observed varying but distinctive nuclear PAK1 localization in parent myxofibrosarcoma cells (**Figure-S6A**), commensurate with significant nuclear PAK1 immunoexpression in a subset of aggressive myxofibrosarcomas detailed below. PAK1 signaling may modulate angiogenesis through multiple pathways, including generation of tumor-derived secreted factors in cancer cells or stimulation of the endothelium-specific PAK1 activation in endothelial cells 13, 19, 25, 26. Our in vitro evidence indicated that CSF2 is a potential PAK1-driven angiogenic factor self-produced by myxofibrosarcoma cells (**Figure-2**). In this context, we interrogated whether and, if so, how PAK1 entered tumoral nuclei to switch on the potential CSF2-mediated angiogenic pathway. As PAK1 falls short of transactivation capability on its own 13, 18, we further examined if the observed PAK1-driven CSF2 overexpression is operated at the transcriptional level and dependent on PAK1 nuclear entry. Interestingly, the *CSF2* mRNA level was significantly downregulated in *shPAK1*-transduced OH931 and NMFH1 cell models but upregulated in NFMH2 cells transfected with pCMV6-PAK1 or pCMV6-PAK1^T423E^, as compared with their corresponding *shLacZ* or empty pCMV6 control (**Figure-2F**). Next, luciferase reporter assays were performed to measure the *CSF2* promoter activity, which was prominently abolished in OH931 and NMFH1 cells stably transduced with either of two *shPAK1* clones. Conversely, the *CSF2* promoter activity in NMFH2 cells was significantly augmented in the transfectants with wild-type PAK1 and those with hyperactivated PAK1^T423E^, being even higher in the latter and upholding a putative transcriptional control (**Figure-2G**).

**Figure 3:**
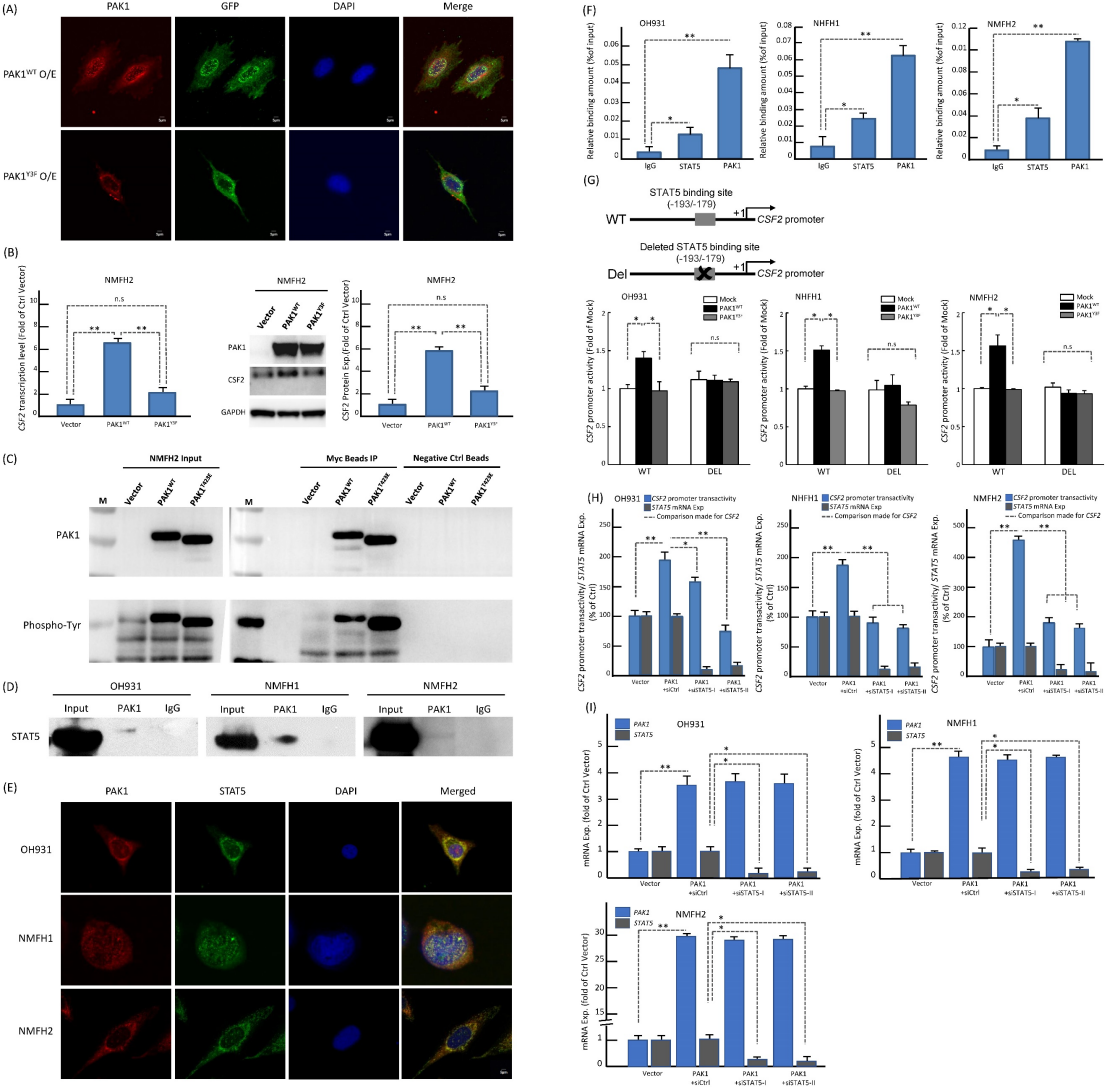
** PAK1 with phosphorylation of both tyrosine and threonine promotes its nuclear entry to co-transactivate *CSF2* via binding to STAT5B in myxofibrosarcoma cells. (A)** Confocal immunofluorescent microscopy demonstrated that pCMV-PAK1 transfection overtly increased the nuclear speckles of PAK1 in NMFH2 cells. In comparison with the pCMV-PAK1 transfectant, pCMV6-PAK1^Y3F^ substantially reduced the nuclear speckles of PAK1 instead, as evidenced by decreased yellow signals in the merged images. *blue* (DAPI nuclear counterstain); *red* (anti-PAK1); *green* (anti-GFP to indicate the successful transfection). **(B)** As compared with the empty control and plotted in histograms, the pCMV-PAK1 NMFH2 transfectant exhibited significantly higher expression levels of *CSF2* mRNA (*left*) and protein (*right*), as determined by qRT-PCR and western blot assays, respectively, while pCMV6-PAK1^Y3F^ significantly negate the CSF2-increasing effect of pCMV-PAK1. **(C)** In the immunoprecipitation assay (IP) using anti-PAK1 as the bait, a significantly greater amount of phosphorylated tyrosines pulled down by anti-PAK1 was observed in the mutant pCMV-PAK1^T423E^ transfectant than in the wild-type pCMV-PAK1 counterpart, indicating that threonine phosphorylation of PAK1 aid in augmenting the tyrosyl phosphorylation essential in PAK1 nuclear entry. (**D-I**) Nuclear PAK1 binds to, co-localizes with, and indispensably requires STAT5B to transactivate *CSF2* in all three parent cell lines. (D) As probed by western blots, the IP eluents showed variable amounts of STAT5B protein pulled down by anti-PAK1, indicating their interaction. (E) The nuclear co-localization of PAK1 (*red*) and STAT5B (*green*) was evidenced by presenting as yellow signals in the merged images using DAPI to counterstain the nuclei. (F) In qChIP assays, there was significantly enriched occupancy of the *CSF2* promoter using either anti-STAT5B or anti-PAK1 as the bait, as compared with IgG serving as the negative control. (G) As indicated in the schematic diagram, the CSF2 promoter constructs with (CSF2-Del) or without (CSF2-WT) deleting the critical STAT5 binding site (-193/-179) were created, co-transfected with empty mock, wild-type pCMV6-PAK1 or pCMV6-PAK1^Y3F^ vector, and then analyzed for the *CSF2* promoter activity. In all three cell lines with co-transfection of CSF2-WT, the higher *CSF2* promoter activity originally driven by wild-type PAK1 was consistently reduced by PAK1^Y3F^ transfection. In contrast, wild-type PAK1 transfection, if co-transfected with CSF2-Del, did not heighten the *CSF2* promoter activity, namely no significant difference between wild-type PAK1 and PAK1^Y3F^ when deleting the STAT5 binding site from the *CSF2* promoter. Concordantly across three cell models, either of both *siSTAT5* clone could significantly decreased the* CSF2* promoter activity (H) and *CSF2* mRNA expression level (I), as compared with the corresponding *siCtrl* controls and plotted in the histograms. *, *P*<0.05; **,* P*<0.01.

Since PAK1 phosphorylation at tyrosine residues 153, 201, and 285 had been shown to dictate nuclear entry 27, we therefore created a PAK1^Y3F^ mutant by site-directed mutagenesis (**Figure-S6B**) and rendered these residues non-phosphorylatable to elucidate its impact on PAK1-driven *CSF2* transactivation in NFMH2 cells stably transfected with pCMV6-PAK1^Y3F^, pCMV6-PAK1 or empty control. As compared with the corresponding parent cell line (**Figure-S6A**), pCMV6-PAK1 overtly increased nuclear relocation of PAK1 in a multifocal speckled pattern, which was substantially negated by pCMV6-PAK1^Y3F^ (**Figure-3A**) under confocal immunoflorescence. Concordantly, pCMV6-PAK1^Y3F^ in NMFH2 cells also significantly decreased *CSF2* expression at the mRNA and protein levels originally achieved by pCMV6-PAK1 (**Figure-3B**). In IP assays of NMFH2 cells, pCMV6-PAK1^T423E^ intriguingly precipitated a greater abundance of total phosphorylated tyrosine than pCMV6-PAK1 (**Figure-3C**). However, in the input NMFH2 lysates, the phosphorylated tyrosine levels in pCMV6-PAK1^T423E^ and pCMV6-PAK1 transfectants were similar, implying the involvement of threonine activation and tyrosyl phosphorylation to facilitate PAK1 nuclear entry in myxofibrosarcomas.

Based on the following reasoning, we posited that STAT5 may play a pivotal role in the functional link between PAK1 nuclear import and *CSF2* transactivation. First, PAK1 with tyrosyl phosphorylation can translocate to the nuclei and promote the activation and nuclear accumulation of STAT5, leading to increased transcriptional activity of STAT5 required for mammary gland development 28 and leukemogenesis 29, 30. Second, the *CSF2* promoter contains the consensus STAT5-binding sequence where phosphorylated STAT5 is known to persistently transactivate *CSF2* gene expression in autoimmune monocytes of type 1 diabetic patients 31. Third, CSF2 signals through JAK2 to induce STAT5 activation during monocyte/macrophage differentiation 32. In three parent lines, co-IP assay for total cell lysates pulled down by anti-PAK1 validated the binding between PAK1 and STAT5B (**Figure-3D**). In contrast, anti-PAK1 did not consistently precipitate STAT5A in every cell line analyzed (**Figure-S6C**). The more essential role of STAT5B in the interaction with nuclear PAK1 was further supported by their speckled nuclear co-localization using confocal immunocytochemistry (**Figure-3E**), implying their direct interaction to promote nuclear entry. Compared with IgG control, anti-PAK1 and anti-STAT5B showed significantly enriched occupancy on the *CSF2* promoter in all parent lines using qChIP assays targeting the STAT5B-binding site (**Figure-3F**)*.* Being consistent in all cell models, the *CSF2* promoter activity was significantly enhanced only when cells were co-transfected with pCMV6-PAK1 and the wild-type *CSF2* promoter construct, but not so in the settings of pCMV6-PAK1^Y3F^ mutant, the deleted *CSF2* promoter construct that nullified STAT5 binding, and both (**Figure-3G**). As compared to the *siCtrl*, transient transduction of either of two *siSTAT5* clones significantly diminished the *CSF2* promoter activity (**Figure-3H**) and mRNA expression (**Figure-3I**) in all cell lines pre-transfected with pCMV6-PAK1. Collectively, these lines of evidence validated the indispensability and cooperation of PAK1 and STAT5B in transactivating *CSF2* to drive angiogenesis.

### Clinical correlations with alterations of PAK1 and CSF2 in myxofibrosarcomas

**Figure 4:**
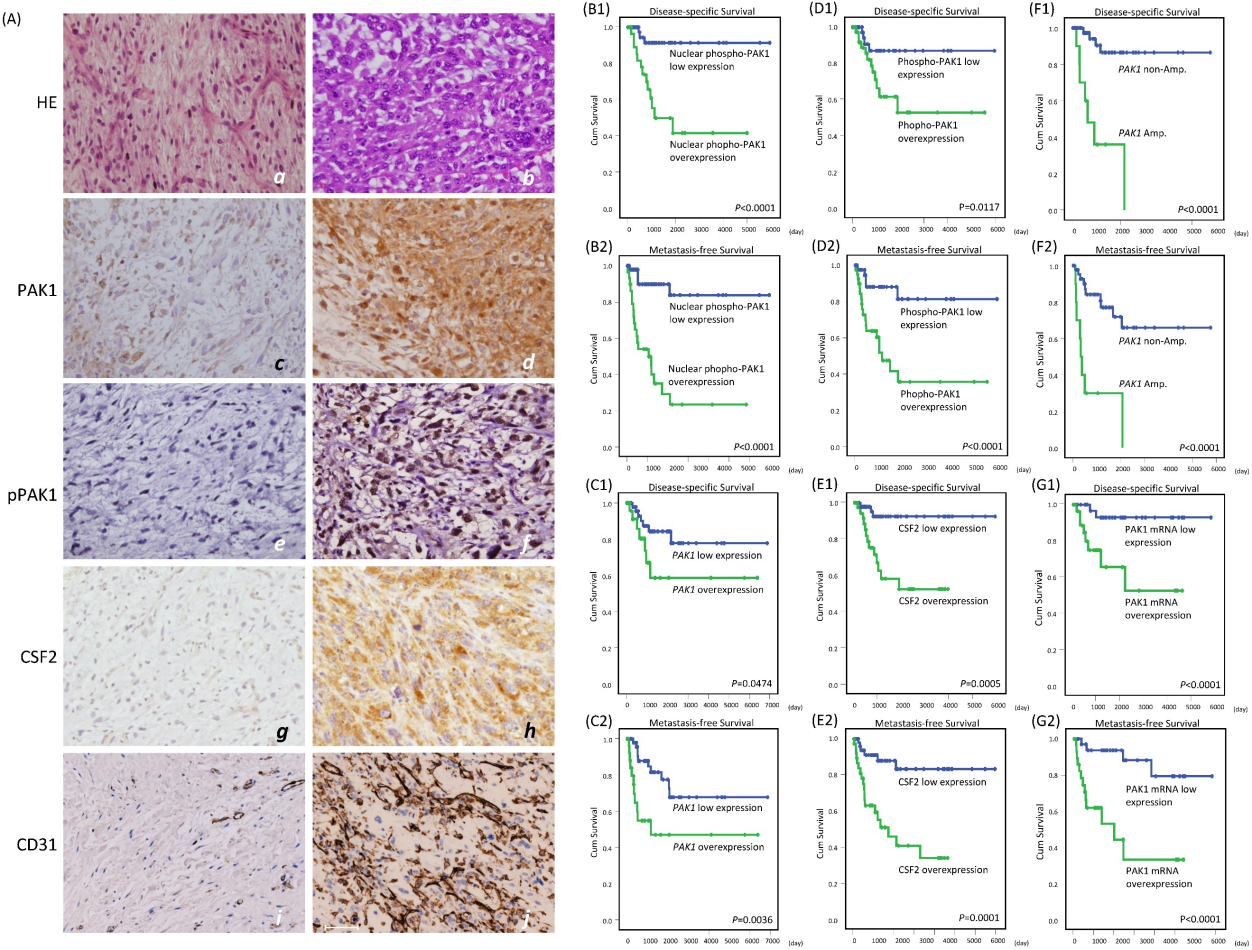
** Immunoexpression levels of whole-cell PAK1, whole-cell and nuclear p-PAK1^T423^, and cytoplasmic CSF2 in low-grade and high-grade myxofibrosarcomas and their correlations with CD31-stained microvascular density and prognosis. (A)** Compared with a grade 1 case (***a***), a grade 3 *(**b**)* myxofibrosarcoma exhibited increased cellularity, nuclear pleomorphism, and mitoses. Expression of whole-cell PAK1 (***c***), labeling of whole-cell and nuclear p-PAK1^T423^ ***(e)***, reactivity of cytoplasmic CSF2 ***(g)***, and CD31-labeled microvessel density (***i***) were focal, low, weak and scarce in the grade 1 case, respectively, while these stains became diffuse (***d***), higher (***f***), stronger (***h***), and denser (***j***) in the grade 3 myxofibrosarcoma. **(B-G)** In univariate prognostic analyses, disease-specific survival and metastasis-free survival were stratified according to the levels of nuclear p-PAK1^T423^ (B_1-2_), whole-cell PAK1 (C_1-2_), whole-cell p-PAK1^T423^ (D_1-2_), cytoplasmic CSF2 (E_1-2_), *PAK1* gene copy status (F_1-2_), and *PAK1* mRNA abundance (G_1-2_).

Using TMA-assembled primary myxofibrosarcoma tissues, we validated the clinical relevance of the *PAK1* gene copy number, *PAK1* mRNA abundance, and immunoexpression levels of whole-cell PAK1, whole-cell and nuclear p-PAK1^T423^, and cytoplasmic CSF2. Immunohistochemical results were informative in 104 cases, including 45 grade 1, 45 grade 2, and 14 grade 3 tumors (**Figure-4A**, **Table-S1**). Whole-cell PAK1, whole p-PAK1^T423^, and cytoplasmic CSF2 were overexpressed in approximately 40% of cases, in which these factors were all positively related to one another (all *P*<0.001) and CD31-labeled MVD (all *P*< 0.02), as quantitated by image J. Overexpressed whole-cell PAK1 and whole-cell p-PAK1^T423^ were also strongly associated with increasing mitoses (both *P*≦0.003), histological grades (both *P*<0.001), and pathological stages (both *P*≦0.009), while CSF2 overexpression was not so. Although nuclear entry was not identified in 10% of cases overexpressing whole-cell p-PAK1^T423^, nuclear p-PAK1^T423^ overexpression was notable in 30% of cases and strongly associated with overexpressed whole-cell PAK1, whole-cell p-PAK1^T423^ and cytoplasmic CSF2 (all *P*<0.001) as well as aforementioned clinicopathological variables (all *P*≦0.007). These findings were in line with in vitro evidence, supporting the pro-angiogenic function of overexpressed/activated PAK1 through nuclear import to co-transactivate *CSF2*.

Among 71 assessable cases (**Table-S1**), *PAK1* amplification preferentially occurred in cases overexpressing whole-cell PAK1, whole-cell p-PAK1^T423^, and nuclear p-PAK1^T423^ (*P*=0.007) and featuring higher mitosis, grades, stages, and MVD (*P*=0.002). *PAK1* mRNA was significantly more abundant in myxofibrosarcomas overexpressing whole-cell PAK1, whole-cell p-PAK1^T423^, nuclear p-PAK1^T423^, and cytoplasmic CSF2 (all *P*≦0.006) and positively associated with the above clinicopathological variables.

In univariate log-rank analyses (**Table-S2**), nuclear p-PAK1^T423^ overexpression (**Figure-4B_1-2_**) strongly predicted shorter DSS (*P* <0.0001) and MeFS (*P* <0.0001, and outperformed overexpressed whole-cell PAK1 (**Figure-4C_1-2_**, DSS, *P* =0.0474; MeFS, *P* =0.0036) and whole-cell p-PAK1^T423^ (**Figure-4D_1-2,_** DSS, *P* =0.0117; MeFS, *P* <0.0001). CSF2 overexpression (**Figure-4E_1-2_**) was notably predictive of shorter DSS (*P*=0.0005) and MeFS (*P*=0.0001), as were higher *FNCLCC* grades, *PAK1* amplification (**Figure-4F_1-2_**, DSS, *P* <0.0001; MeFS, *P* <0.0001) and high *PAK1* mRNA abundance (**Figure-4G_1-2_**, DSS, *P* <0.0001; MeFS, *P* <0.0001). AJCC stage 3 was only associated with shorter MeFS (*P* =0.0181). In multivariate comparisons (**Table-S3**), CSF2 overexpression was significantly predictive of shorter DSS (*P*=0.014, hazard ratio=6.219), while whole-cell p-PAK1^T423^ overexpression effectively portended shorter MeFS (*P*=0.018, hazard ratio=3.992). Notably, higher *FNCLCC* grades remained prognostically independent as an adverse factor (*P*<0.001 for both survival endpoints), whereas AJCC stage 3 disease lost significance.

### Pan-PAK inhibitor, PF-3758309, suppressed PAK1-expressing myxofibrosarcoma cells through induction of apoptosis and inhibition on angiogenesis

**Figure 5:**
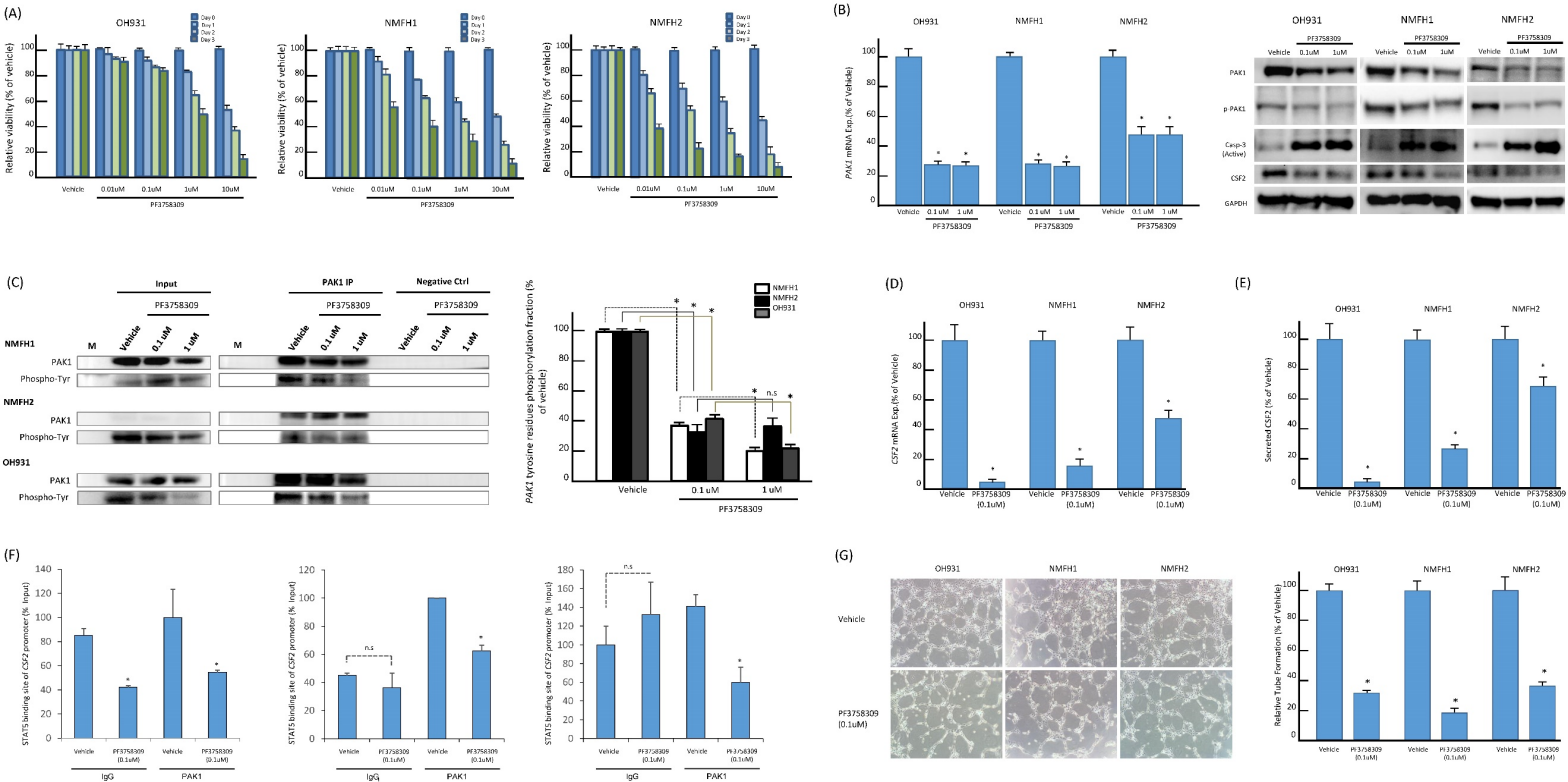
** In vitro, PF3758309 effectively inhibited cell viability and PAK1 expression and phosphorylation, induced cell apoptosis, and abrogated angiogenesis via downregulating STAT5B-mediated *CSF2* transactivation. (A)** As indicated by the histograms, NMFH2 and NMFH1 cell lines were more susceptible to treatment than OH931 cells, given that the former two exhibited a reduction of viable cells by nearly or over 50% when incubated with PF3758309 for 72h at 0.01 μM. However, a similar inhibitory effect on viability at 72h was achieved at 1 μM in OH931 cells. **(B-C)** PF3758309 suppressed expression and phosphorylation of PAK1 in vitro. In qRT-PCR assay (B, *left panel*), PF3758309, both at 0.1 μM and 1 μM as compared with vehicle control, significantly downregulated the abundance of *PAK1* mRNA in all three parent cell lines. In western blots of three myxofibrosarcoma cell models (B, *right panel*), PF3758309, dose-dependently in general at indicated doses, decreased the levels of phosphorylated PAK1 threonines and, to a lesser degree, those of the total PAK1, concomitantly accompanied by decreased abundance of CSF2 and increased expression of cleaved caspase-3, indicative of apoptosis. In the anti-PAK1 IP assays (C), the phosphorylated tyrosine levels of PAK1 were significantly attenuated in all three parent cell lines analyzed. The dose-dependent suppressive effect of PF3758309 was also observed in PAK1-overexpressing NMFH1 and OH931 cell models, as depicted in the histogram *(right)*. **(D-F)** Compared with vehicle controls, PF3758309 significantly suppressed *CSF2* transactivation and expression via decreasing the binding to STAT5B. The levels of *CSF2* mRNA abundance by qRT-PCR (D), secreted* CSF2* protein by ELISA (E), and the occupancy of *CSF2* promoter at the STAT5B binding site in anti-PAK1 ChIP assays (F) were consistently diminished upon exposure to PF3758309 for 72h at 0.1μM across all three cell lines. **(G)** In HEVEC-based angiogenic assays, PF3758309 at 0.1μM remarkably abolished the tube-forming capacity of endothelial cells in all three cell lines as plotted in the histograms. All in vitro assays were performed in triplicate and presented as the mean ± SD. *, *P*<0.05.

PF-3758309, a potent ATP-competitive pyrrolopyrazole pan-PAK inhibitor, was reported to inhibit carcinomas, melanomas, and rhabdomyosarcomas by abrogating interactions of PAK1 and PAK4 with their corresponding substrates 14, 15, 33. These results prompted us to assess the in vitro effects of PF-3758309 on the myxofibrosarcoma cells exhibiting overexpressed PAK1 as a potentially druggable target. PF-3758309, incubated for 72 h at indicated doses, exhibited significant inhibition on cell viability of all parent cell lines in XTT assays, while their susceptibility varied with corresponding IC_50_ values from 0.01 μM (NMFH-1, NMFH2) to 1 μM (OH931) (**Figure-5A**). In addition, the *PAK1* mRNA levels were significantly reduced by PF-3758309, both at 0.1 μM and 1 μM. In a dose-dependent manner, treatment with PF-3758309 decreased the protein levels of total PAK1, phosphorylated threonines of PAK1, and CSF2 but increased expression of cleaved caspase 3, indicative of induced apoptosis (**Figure-5B**). The anti-PAK1 IP assay also demonstrated significant abrogation of phosphorylated tyrosines upon PF-3758309 treatment, which was consistent across all three cell lines and even exhibited significant dose-dependent differences in PAK1-overexpressing NMFH1 and OH931 cell lines (**Figure-5C**). As evidenced by increased subG_0-1_ populations and Annexin V-stained apoptotic/necrotic cells, flow cytometry substantiated that PF-3758309-treated parent cell lines underwent apoptosis to varying extent and arrested cell cycle progression variably at G1, G1/S or G2/M phase (**Figures-S7A-C**). In all PF-3758309-treated parent myxofibrosarcoma cell lines relative to vehicle controls, PF-3758309 at 0.1 μM significantly abolished the levels of *CFS2* mRNA (**Figure-5D**) and secreted CFS2 protein (**Figure-5E**), the PAK1 occupancy at the STAT5B binding site of the *CSF2* promoter in the ChIP assay (**Figure-5F**), and the angiogenic capacity in the HUVEC assay (**Figure-5G**).

### CSF2-mediated oncogenic effect of PAK1 ovexpression in vivo

NMFH2 xenografts with ectopic PAK1 ovexpression and *siCtrl* exhibited larger average tumor volumes as per caliper measurement, which became significant from Day 10 post-implantation onward and kept this trend until Day 19, as compared with the group with empty vector and *siCtrl* (**Figure-S8A**). Notably, this in vivo tumor-promoting effects of PAK1 in myxofibrosarcoma was effectively negated upon transduction with *siCSF2* in the PAK1-transfected mice, even to the degree indistinguishable from that of the empty control/*siCtrl* group, as evidenced by gross examination of excised xenografts (**Figure-S8A-B**). Histologically, the PAK1/*siCtrl* NMFH2 xenografts displayed the highest cellularity of proliferative pleomorphic cells with very scant intervening stroma (**Figure-S8C**). However, relatively lower cellularity was observed in the mice of both empty control/*siCtrl* and PAK1/*siCSF2* groups, with more apparently increased collagenous matrix in the latter. The significantly increased nuclear and cytoplasmic PAK1 expression exquisitely exemplified successful PAK1 transfection in both PAK1-transfected groups, irrespective of *siCtrl* or *siCSF2* transduction. Notably, CSF2 expression was present only in the PAK1/*siCtrl* group, accompanied by higher CD31-stained MVD and Ki-67 proliferative index which were overtly lowered in both the empty control/*siCtrl* and PAK1/*siCSF2* groups **(Figure-S8C)**.

### Therapeutic effect of PF-3758309 in myxofibrosarcoma xenografts

**Figure 6:**
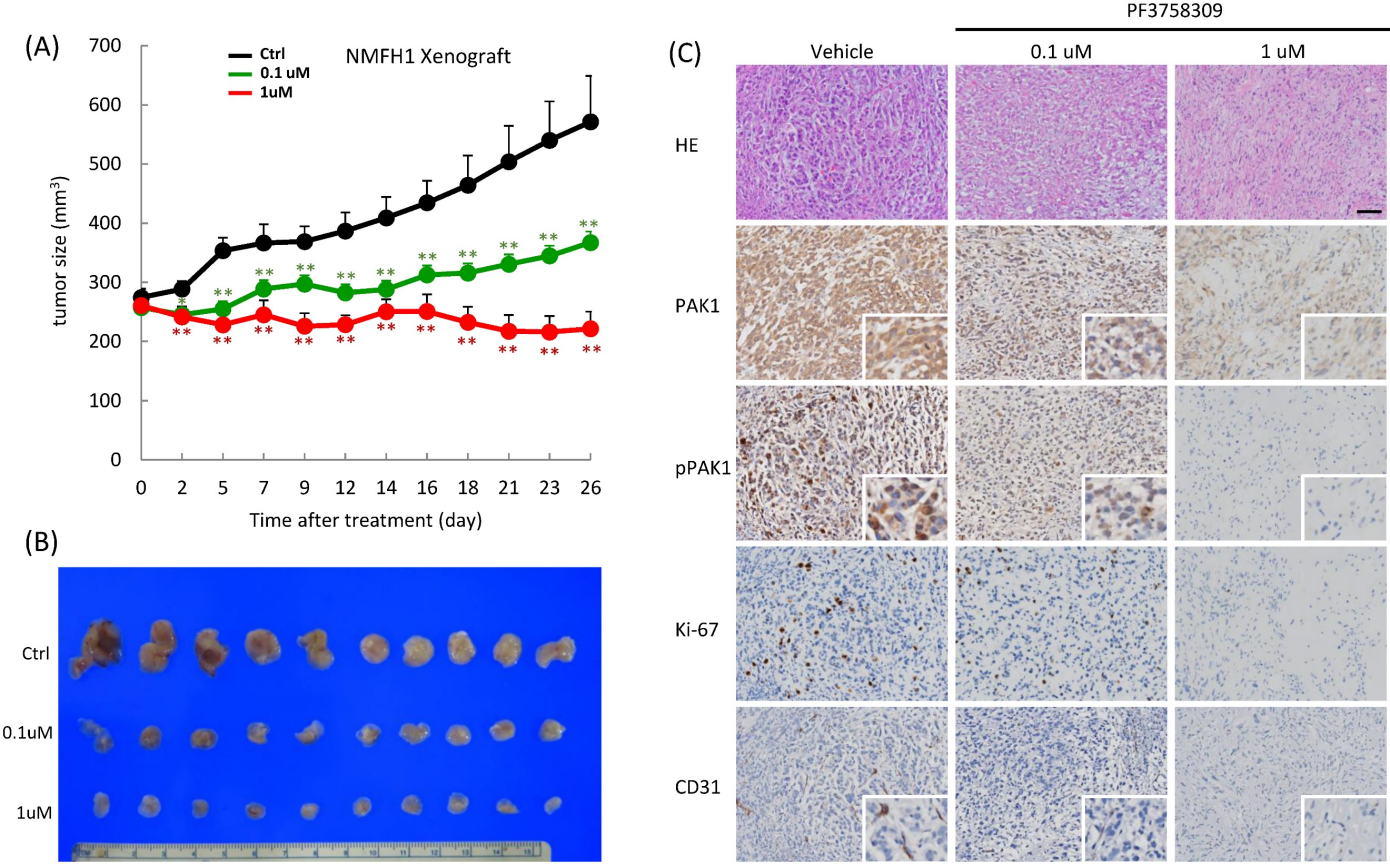
** PF3758309 induced dose-dependent suppression of tumor growth in NMFH1 xenografts. (A)** As plotted in the caliper-measured growth curves (*upper,* n=10 in each group), the mean tumor volumes in the groups of mice treated with PF3758309 at 1 μM (*P*=0.003) or at 0.1 μM (*P*=0.012) both became significantly smaller on Day 2 and retained these significant differences until Day 26 post-treatment, in comparison with the group with vehicle control. Note that a significant dose-dependent difference in the growth-inhibitory effect of PF3758309 was also noted between 1 μM and 0.1 μM (*P*=0.001).** (B)** When the mice were sacrificed on Day 26, xenografts treated with vehicle controls exhibited the largest gross sizes, followed by those with PF3758309 at 0.1 μM and then by those at 1 μM. **(C)** Compared with vehicle controls, xenografts treated with PF3758309 at 0.1 μM and 1.0 μM displayed, in a dose-dependent manner, less prominent tumor pleomorphism and cellularity, decreased expression levels of whole-cell PAK1, whole-cell and nuclear phospho-PAK^T423^ and Ki-67 proliferative index, and attenuated microvascular density by counting CD31-stained tumor vessels. Insets; higher magnifications of representative foci. *, *P*<0.05; **, *P*<0.01.

In vivo, PF-3758309 exhibited apparent growth inhibition on NMFH1 xenografts, which, compared with the PBS-treated vehicle controls, became significant post-treatment on Day 2, exhibited significant dose-dependent difference between groups receiving 0.1 μM and 1 μM on Day 7, and kept these significant differences until sacrifice on Day 26 (**Figure-6A-B**). Histologically, the vehicle-treated xenografts displayed myxoid reticular to solid hypercellular growth of high-grade bizarre cells with frequent mitotic activity. In contrast, PF-3758309 treatment elicited histological regression with decreased nuclear grading at 0.1μM and caused hypocellular proliferation of low-grade fibroblastic cells with nuclear spindling in collagenous matrix at 1μM. Notably, PF-3758309-induced morphologic alterations were dose-dependently associated with the decreased levels of whole-cell PAK1, whole-cell and nuclear p-PAK1^T423^, Ki-67 and CD31-stained MVD (**Figure-6C**).

## Discussion

Deregulation of various PAKs may perturb pleotropic cellular processes, hence contributing to tumor development and progression 13, 19, 33. Apart from validating the canonical pro-proliferative and pro-metastatic functions of PAK1, we have characterized a hitherto undescribed PAK1/STAT5B/CSF2 regulatory axis in myxofibrosarcomas, wherein the pro-angiogenic attribute of PAK1 offers a viable molecular target to inform risk stratification and therapeutic strategies. Although PAK1-associated oncogenesis is thought to classically depend on the interaction of PAK1 with RhoGTPases in the cytosol 13, 17, 19, 34, 35, PAK1 nuclear entry has emerged as a crucial mechanism to regulate expression of myriad downstream cancer-associated genes 12, 13, 18, 19, 36. In our cell-based models, concomitant tyrosyl and threonine phosphorylation expedited the nuclear entry of activated PAK1, through which PAK1 interacted with nuclear STAT5B at the STAT5 binding site to co-transactivate the *CSF2* promoter. The targetability of PAK1-driven angiogenesis, not linked to VEGF or VEGF-C, was demonstrated by knockdown of involved molecules in the pro-angiogenic axis and pharmacologic inhibition of PAK1 with PF3758309 in vitro, as well as significant inhibition on MVD and tumor growth by either *siCSF2* or PF3758309 in vivo.

To varying degrees of statistical significance, *PAK1* amplification, CSF2 overexpression, and aberrancies in PAK1 expression, activation, and nuclear localization were all significantly related to one another and associated with higher *FNCLCC* grades, MVD, and, more importantly, shorter univariate DSS and MeFS.

These robust associations not only highlighted the clinical and therapeutic relevance of PAK1-driven angiogenesis in myxofibrosarcomas but also mirrored the contributions of overexpressed, activated, and nuclear PAK1 in promoting tumor growth, migration/invasion, and angiogenesis observed in vitro and in vivo. Of notice, these characteristics provided a rationale to identify aggressive myxofibrosarcomas, which were significantly *PAK1*-amplified and potentially actionable due to the intrinsic genetic vulnerability in angiogenesis. Since over half of high expressers of whole-cell PAK1 and whole-cell p-PAK1 (**Table-S1**) were not *PAK1*-amplified, a larger proportion of PAK1-deregulatd myxofibrosarcomas might harbor non-amplified mechanisms, given that post-transcriptional control, such as non-coding mRNAs or alternative polyadenylation, are increasingly known to operate in various cancers to drive PAK1 expression and/or activation 37, 38. For instance, activation of PAK1 in Ewing sarcomas was found attributable to aberrantly overexpressed miR-130b, which directly downregulates ARHGAP1 and leads to activation of downstream CDC42/PAK1/JNK/AP1 cascades 39.

Signal transduction-induced cytokine surge can be modulated at multiple levels by kinases and their adaptors, interacting partners, and nuclear relocation after activation 40. In our myxofibrosarcoma models, PAK1 nuclear entry significantly increased STAT5B transcriptional output to enhance* CSF2* transactivation, as evidenced by the direct interaction between PAK1 and STAT5B, the *CSF2* promoter occupancy by STAT5B/PAK1, and the negative impact on *CSF2* promoter luciferase activity imposed by the STAT5B binding site-deleted construct and *siSTAT5*. Notably, pCMV6-PAK1^Y3F^ significantly downregulated *CSF2* mRNA and protein, and both *siCSF2* and neutralizing anti-CSF2 effectively abolished PAK1-induced HUVEV network formation. Furthermore, *siCSF2* transduction, as compared with* siCtrl*, could convincingly abrogate the CD31-stained MVD in PAK1-transfected xenografts, hence upholding that PAK1 overexpression/hyperactivation enables *CSF2* transactivation and increases CSF2 secretion to promote angiogenesis.

The above findings indicated the essentiality of PAK1 nuclear entry in the transcriptional upregulation of* CSF2*, in turn promoting secretion of this angiogenic cytokine into tumor microenvironment. Recently, the transcriptional cooperation between nuclear PAK1 and various STAT members has been mechanistically linked to underlie mammary gland development 28, breast cancer stem cell formation 41, and pathogenesis of several leukemic subtypes 29, 30. In these physiologic or pathologic processes, JAK2 is a notable upstream kinase, among others, which enables PAK1 activation and nuclear localization 27, 42-44. Following radiation to lung cancers, PAK1 can be phosphorylated by JAK2 at critical residues of tyrosine, instead of serine/threonine, to maintain protein stability and enhance nuclear translocation 45. This JAK2-mediated PAK1 tyrosyl phosphorylation strengthens the transcriptional repression activity of snail to mediate epithelial-mesenchymal transition and radioresistance, while PAK1^Y3F^ mutant nullifies these effects in irradiated lung cancers 45. The nuanced difference in the mode of PAK1 phosphorylation between myxofibrosarcomas and irradiated lung cancers is probably cellular context-dependent. In myxofibrosarcoma cell models, the constitutively hyperactive PAK1^T423E^ significantly increased the PAK1 tyrosine phosphorylation and PF3758309 enabled concomitant inhibition on the phosphorylated threonine and tyrosine of PAK1. These findings seemingly indicated functional synergy between phosphorylated tyrosines and phosphorylated threonines. Interestingly, increased CSF2 expression in myeloid cells of non-obese diabetic mice may augment its own transcription in an autocrine manner through increasing STAT5 binding to the *CSF2* promoter 46. It has been demonstrated that CSF2 may induce angiogenesis through in vivo activation of JAK2/STAT3 pathway 47. These clues further infer a question of whether, in a positive feedback loop, overproduced CSF2 in myxofibrosarcoma cells depends on nuclear PAK1 to invigorate the activity of JAK2/STAT5B cascade. Moreover, aside from pro-angiogenic function, CSF2 is increasingly recognized to exacerbate tumor progression by its cytokine-mediated immunosuppressive effects on various surrounding cells in tumor microenvironments 48, hence leading to sheer reliance on CSF2 for tumor growth in several solid cancers with adverse prognostic impact. In this context, it is sobering to rethink the adjuvant administration of recombinant CSF2 to ameliorate therapy-induced neutropenia in CSF2-releasing cancers 49, such as myxofibrosarcomas with overexpressed and activated PAK1, which is at risk of detrimental cancer-promoting effect.

In a variety of human malignancies, overexpression of activated PAK1 is known to associate with 'addiction' to this kinase, rendering increased sensitivity to PAK inhibition, especially in *PAK1*-amplified cancers 19. Additionally, PAK1 inhibition is plausible for cancers in which PAK1 converges crucial signaling transductions of aberrated oncogenes (e.g, *ErbB2*, *TRIO*) or tumor suppressor genes (e,g *NF1*) 13, 34, 35. Relevantly, amplified and translocated *TRIO* and loss-of-function *NF1* mutation have been unveiled in subsets of myxofibrosarcomas 5, 6, 8, 50, 51. Through conferring the potent pro-angiogenic capacity, PAK1 overexpression/activation may represent a viable druggable target of myxofibrosarcomas. In vitro, genetic ablations of PAK1 and CSF2, the CSF2-directed antibody, and pharmacological intervention with PF-3758309 inhibitor all significantly abrogated angiogenesis of myxofibrosarcoma. Besides curbing expression and phosphorylated tyrosine and threonine of PAK1, PF-3758309 was functionally validated to downregulate the STAT5 binding to* CSF2* promoter with concomitantly decreased* CSF2* mRNA level and protein secretion. Although PF3758309 is considered a pan-PAK inhibitor not targeting PAK1 alone 14, 33, this drug might indeed exert a notably specific repressive effect on PAK1-driven angiogenesis, an oncogenic attribute not redundantly phenocopied by PAK2-4, in myxofibrosarcomas at least. In NMFH1 xenografts, PF3758309 treatment dose-dependently decreased the expression levels of whole-cell PAK1 and nuclear p-PAK1 and correspondingly led to lower Ki-67 index and MVD, hence achieving significant tumor growth inhibition. The in vivo anti-proliferative and anti-angiogenic effects of PF3758309, largely recapitulating those seen in the group of PAK1/*siCSF2* mice, further underscore the crucial therapeutic relevance of deregulated PAK1. Notably, the PAK1/CSF2 pro-angiogenic axis is not mechanistically linked to the VEGF and VEGF-C. Nevertheless, this result does not completely exclude the possibility of VEGF inhibitors as a therapeutic strategy in myxofibrosarcomas, although pazopanib, a multi-kinase inhibitor also curbing VEGF-VEGFR axis, only showed limited efficacy 52. Moreover, no prospective randomized clinical trials have systematically assessed the effect of VEGF inhibition in myxofibrosarcomas. Collectively, our findings shed promising light on future management of myxofibrosarcomas with the vulnerability of PAK1-driven angiogenesis, either alone or in combination with conventional chemotherapy.

## Conclusions

Overexpressed PAK1 is recurrently amplification-driven in myxofibrosarcomas in which it confers pro-proliferative, pro-metastatic, and pro-angiogenic phenotypes. To promote angiogenesis, PAK1 undergoes tyrosyl and threonine phosphorylation to facilitate its nuclear entry, along with nuclear STAT5B, to co-transactivate *CSF2*, resulting in increased CSF2 secretion. Our findings provide not only prognostic insight into future risk stratification but also a novel biomarker-guided therapeutic approach to targeting angiogenesis in aggressive myxofibrosarcomas which harbor the vulnerable PAK1/STAT5B/CSF2 proangiogenic axis.

## Supplementary Material

Supplementary figures and tables, methods.

## Abbreviations

AJCC: American joint committee on cancer
BrdU: bromodeoxyuridine
CAN: copy number alteration
Co-IP: Co-immunoprecipitation
CSF2: colony stimulating factor 2 (also known as GM-CSF: granulocyte-macrophage colony stimulating factor)
DSS: disease-specific survival
FISH: fluorescence in situ hybridization
FNCLCC: Fédération Nationale des Centres de Lutte Contre Le Cancer
HUVEC: human umbilical venous endothelial cells
LI: labeling index
MeFS: metastasis-free survival
MVD: microvascular density
PAK1: p21 (RAC1) activated kinase 1
p-PAK1^T423^: phosphorylated PAK1 at threonine 423
qRT-PCR: quantitative reverse-transcription polymerase chain reaction
qChIP: quantitative chromatin immunoprecipitation
RSF1: remodeling and spacing factor 1
CEBP/β: CCAAT enhancer binding protein beta
SCID: severe combined immunodeficiency
STAT5A: signal transducer and activator of transcription 5A
STAT5B: signal transducer and activator of transcription 5B
TMA: tissue microarray
