# Supplementary material for: PAK1 overexpression promotes myxofibrosarcoma angiogenesis through STAT5B-mediated CSF2 transactivation: clinical and therapeutic relevance of amplification and nuclear entry
Source: Int J Biol Sci. 2023 Jul 31;19(12):3920–36. doi: 10.7150/ijbs.83467 (PMC10411477; doi:10.7150/ijbs.83467)
Supplement: Supplementary file 1 — Supplementary figures and tables, methods. [file ijbsv19p3920s1.pdf]

Figure-S1

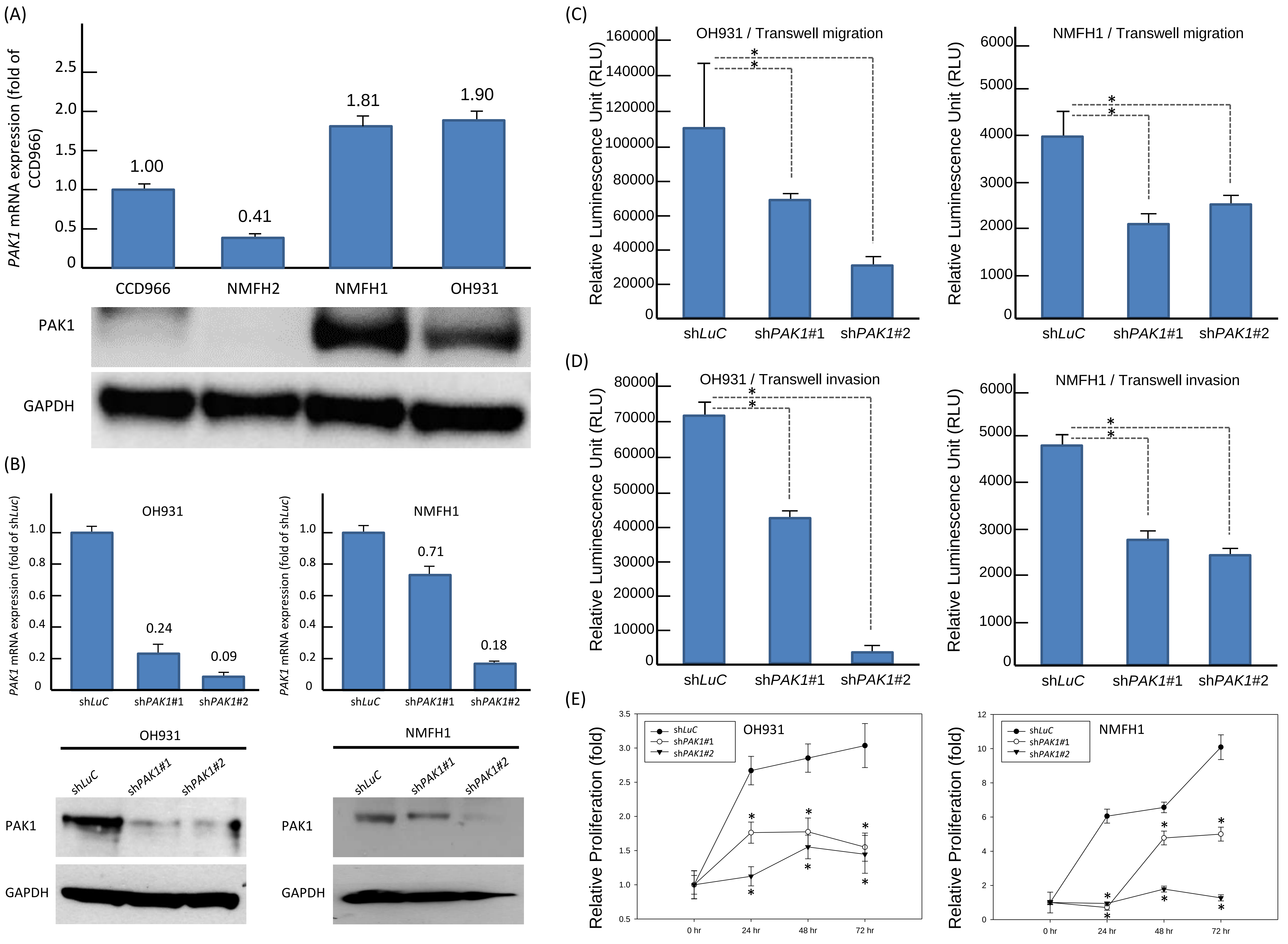
(A)
PAK1 mRNA expression (fold of CCD966)
1.00
0.41
1.81
1.90
CCD966
NMFH2
NMFH1
OH931
PAK1
GAPDH
(B)
OH931
NMFH1
PAK1 mRNA expression (fold of shLuc)
0.24
0.09
0.71
0.18
shLuC
shPAK1#1
shPAK1#2
PAK1
GAPDH
(C)
OH931 / Transwell migration
NMFH1 / Transwell migration
Relative Luminescence Unit (RLU)
(D)
OH931 / Transwell invasion
NMFH1 / Transwell invasion
(E)
Relative Proliferation (fold)
0 hr
24 hr
48 hr
72 hr

Figure-S2

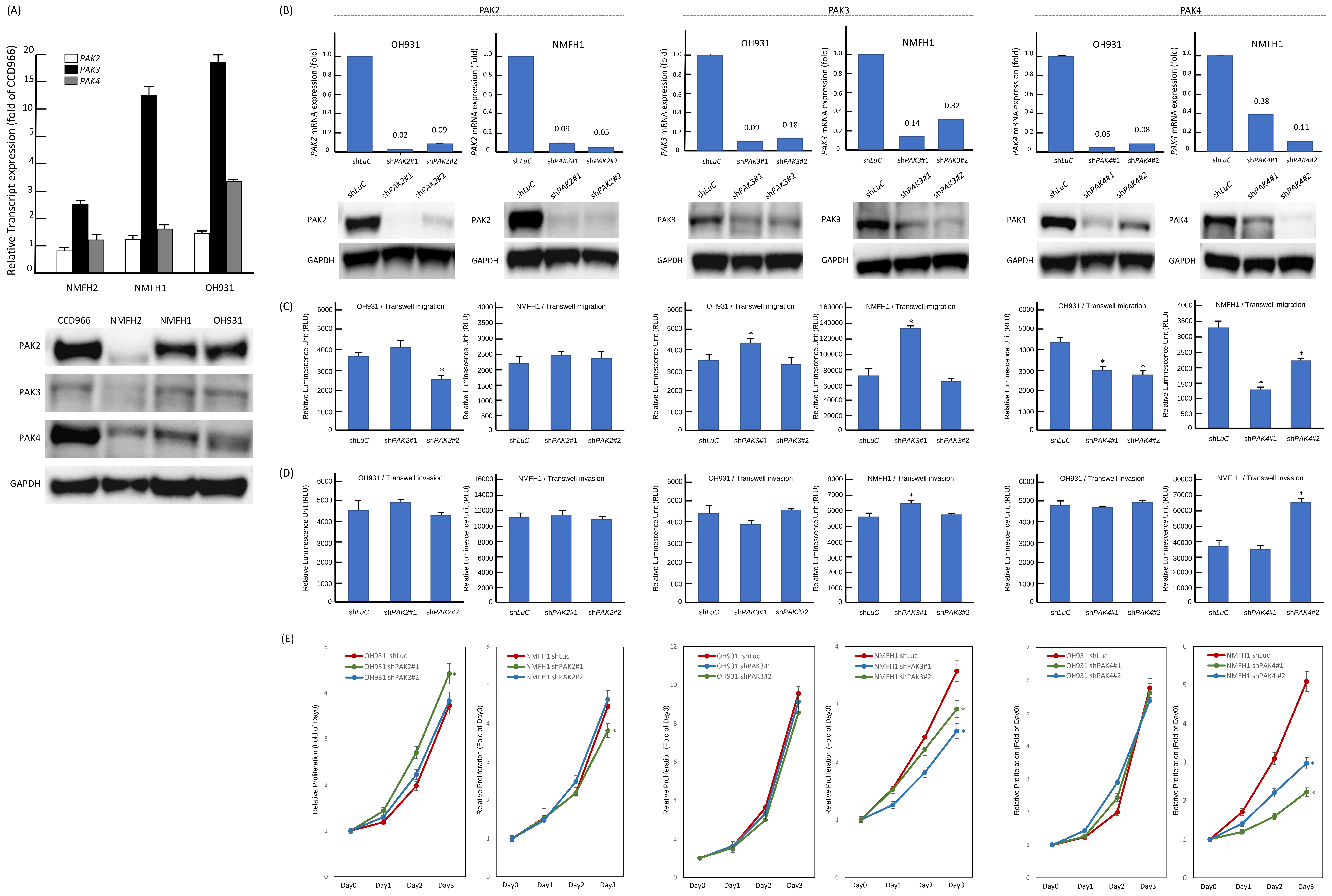

(A)
Relative Transcript expression (fold of CCD966)
PAK2
PAK3
PAK4
NMFH2
NMFH1
OH931
CCD966
GAPDH
(B)
PAK2
PAK3
PAK4
OH931
NMFH1
PAK2 mRNA expression (fold)
PAK3 mRNA expression (fold)
PAK4 mRNA expression (fold)
shLuC
shPAK2#1
shPAK2#2
shPAK3#1
shPAK3#2
shPAK4#1
shPAK4#2
(C)
OH931 / Transwell migration
NMFH1 / Transwell migration
Relative Luminescence Unit (RLU)
(D)
OH931 / Transwell invasion
NMFH1 / Transwell invasion
(E)
OH931 shLuc
OH931 shPAK2#1
OH931 shPAK2#2
NMFH1 shLuc
NMFH1 shPAK2#1
NMFH1 shPAK2#2
OH931 shPAK3#1
OH931 shPAK3#2
NMFH1 shPAK3#1
NMFH1 shPAK3#2
OH931 shPAK4#1
OH931 shPAK4#2
NMFH1 shPAK4#1
NMFH1 shPAK4 #2
Relative Proliferation (Fold of Day0)
Day0
Day1
Day2
Day3

Figure-S3

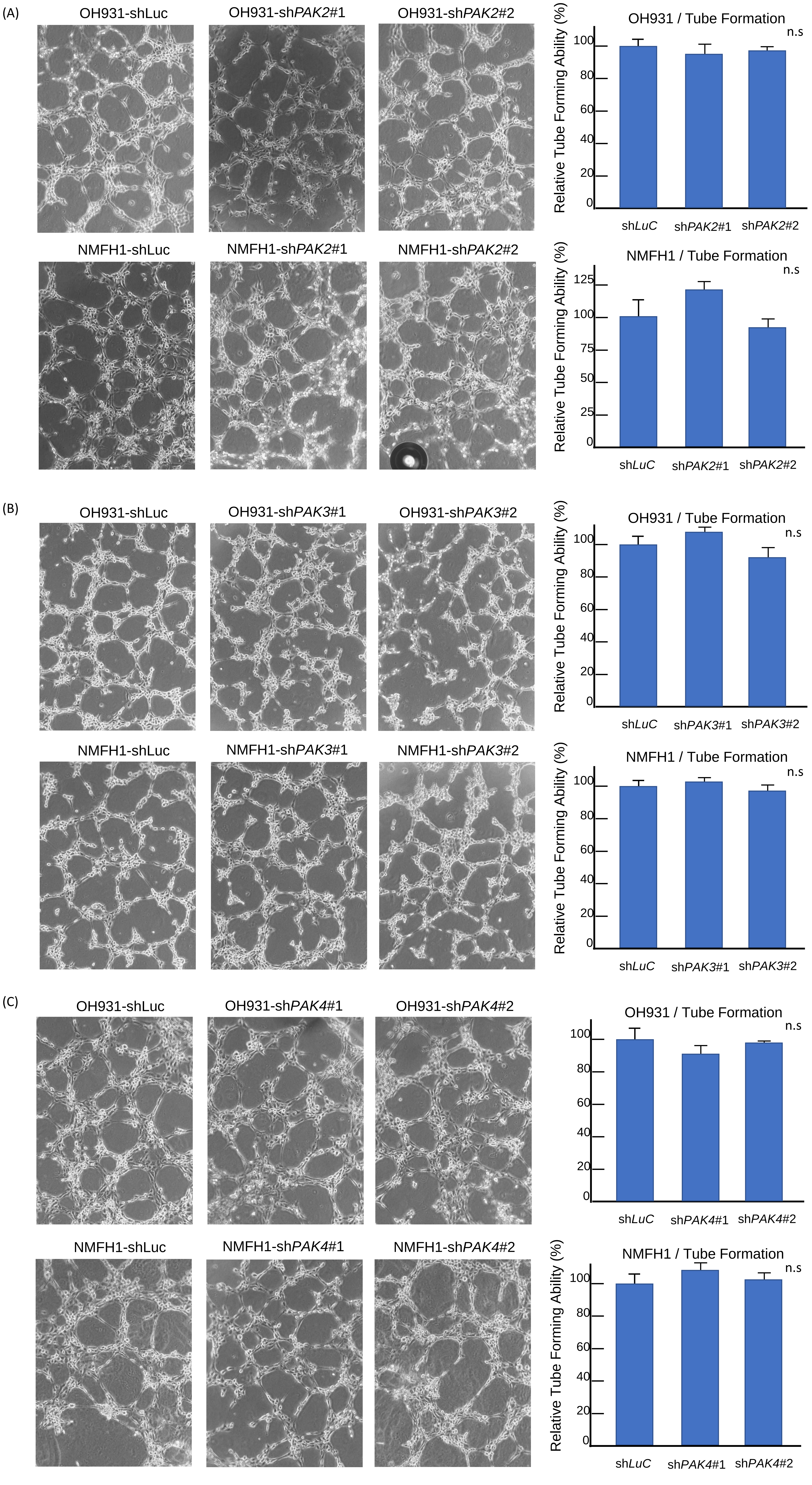
(A)
OH931-shLuc
OH931-shPAK2#1
OH931-shPAK2#2
OH931 / Tube Formation
n.s
Relative Tube Forming Ability (%)
0
20
40
60
80
100
shLuC
shPAK2#1
shPAK2#2
NMFH1-shLuc
NMFH1-shPAK2#1
NMFH1-shPAK2#2
NMFH1 / Tube Formation
n.s
Relative Tube Forming Ability (%)
0
25
50
75
100
125
shLuC
shPAK2#1
shPAK2#2
(B)
OH931-shLuc
OH931-shPAK3#1
OH931-shPAK3#2
OH931 / Tube Formation
n.s
Relative Tube Forming Ability (%)
0
20
40
60
80
100
shLuC
shPAK3#1
shPAK3#2
NMFH1-shLuc
NMFH1-shPAK3#1
NMFH1-shPAK3#2
NMFH1 / Tube Formation
n.s
Relative Tube Forming Ability (%)
0
20
40
60
80
100
shLuC
shPAK3#1
shPAK3#2
(C)
OH931-shLuc
OH931-shPAK4#1
OH931-shPAK4#2
OH931 / Tube Formation
n.s
0
20
40
60
80
100
shLuC
shPAK4#1
shPAK4#2
NMFH1-shLuc
NMFH1-shPAK4#1
NMFH1-shPAK4#2
NMFH1 / Tube Formation
n.s
Relative Tube Forming Ability (%)
0
20
40
60
80
100
shLuC
shPAK4#1
shPAK4#2

Figure-S4

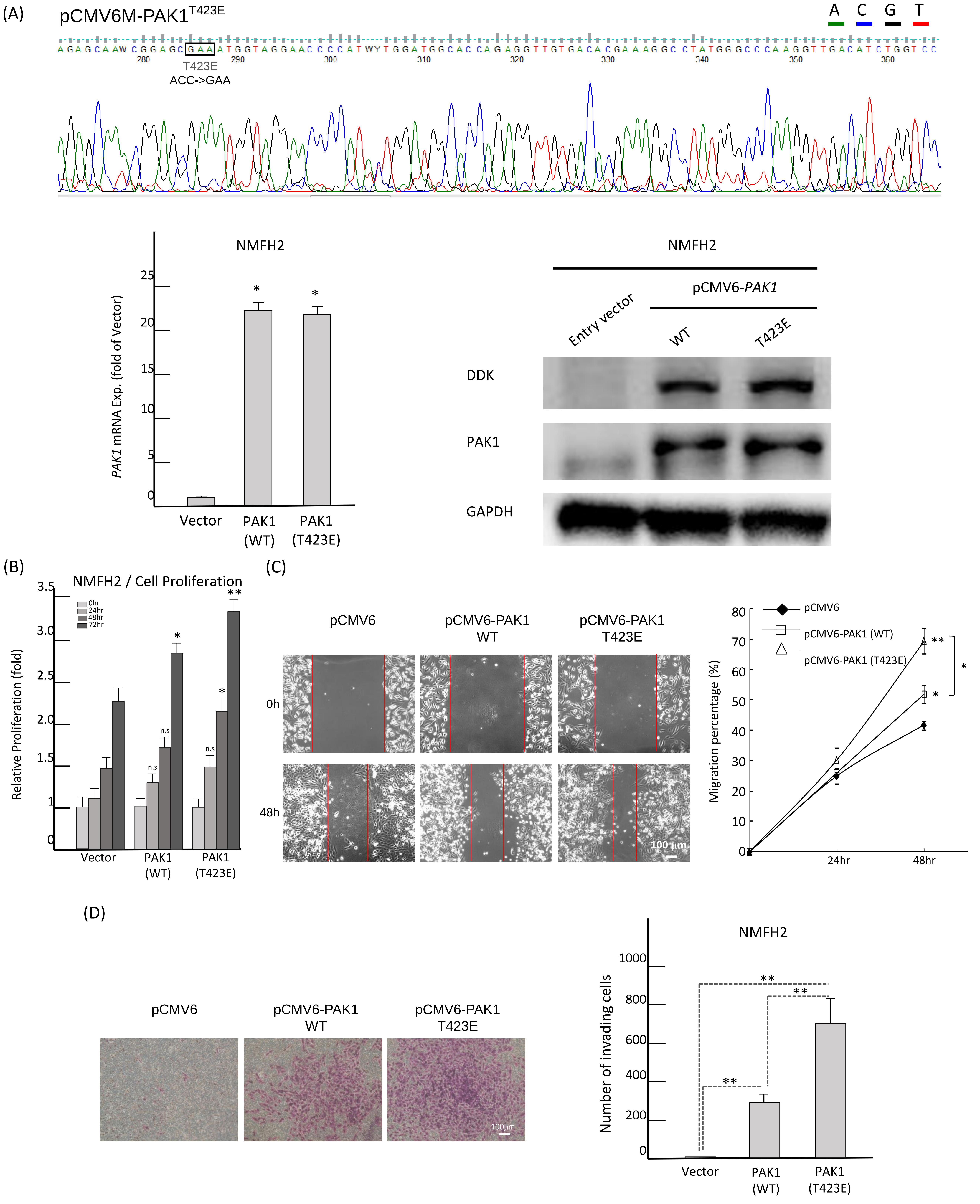
(A)
pCMV6M-PAK1T423E
A C G T
280 290 300 310 320 330 340 350 360
T423E
ACC->GAA
NMFH2
PAK1 mRNA Exp. (fold of Vector)
Vector
PAK1 (WT)
PAK1 (T423E)
NMFH2
pCMV6-PAK1
Entry vector
WT
T423E
DDK
PAK1
GAPDH
(B)
NMFH2 / Cell Proliferation
0hr
24hr
48hr
72hr
Relative Proliferation (fold)
n.s
Vector
PAK1 (WT)
PAK1 (T423E)
(C)
pCMV6
pCMV6-PAK1 WT
pCMV6-PAK1 T423E
0h
48h
100 µm
pCMV6
pCMV6-PAK1 (WT)
pCMV6-PAK1 (T423E)
Migration percentage (%)
24hr
48hr
(D)
pCMV6
pCMV6-PAK1 WT
pCMV6-PAK1 T423E
100µm
NMFH2
Number of invading cells
Vector
PAK1 (WT)
PAK1 (T423E)

Figure-S5

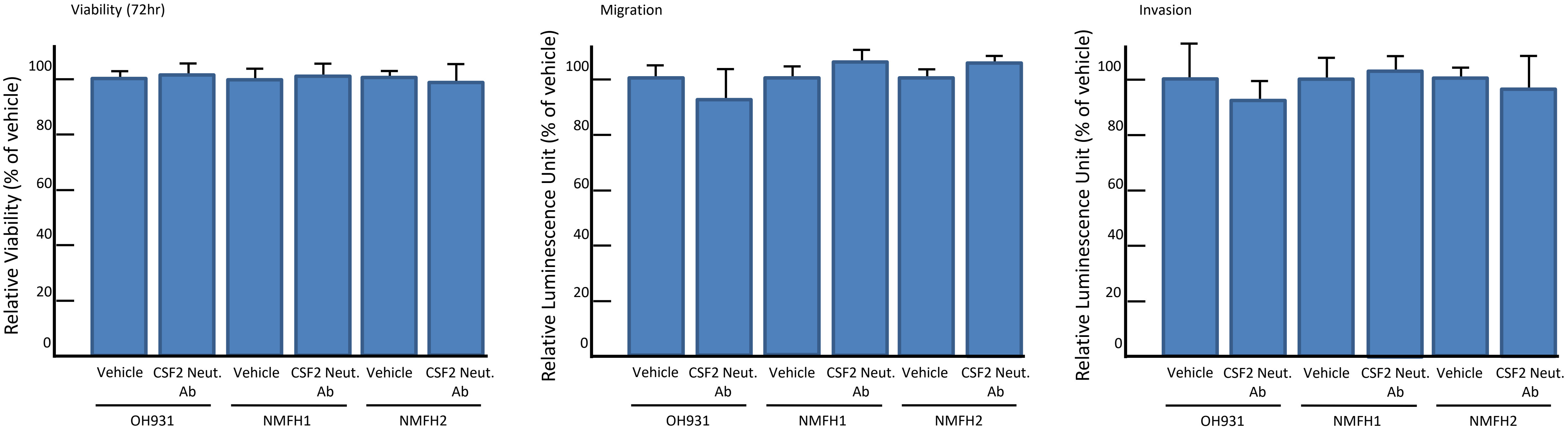
Viability (72hr)
Relative Viability (% of vehicle)
Migration
Relative Luminescence Unit (% of vehicle)
Invasion
Relative Luminescence Unit (% of vehicle)
0
20
40
60
80
100
Vehicle
CSF2 Neut. Ab
OH931
NMFH1
NMFH2

Figure-S6

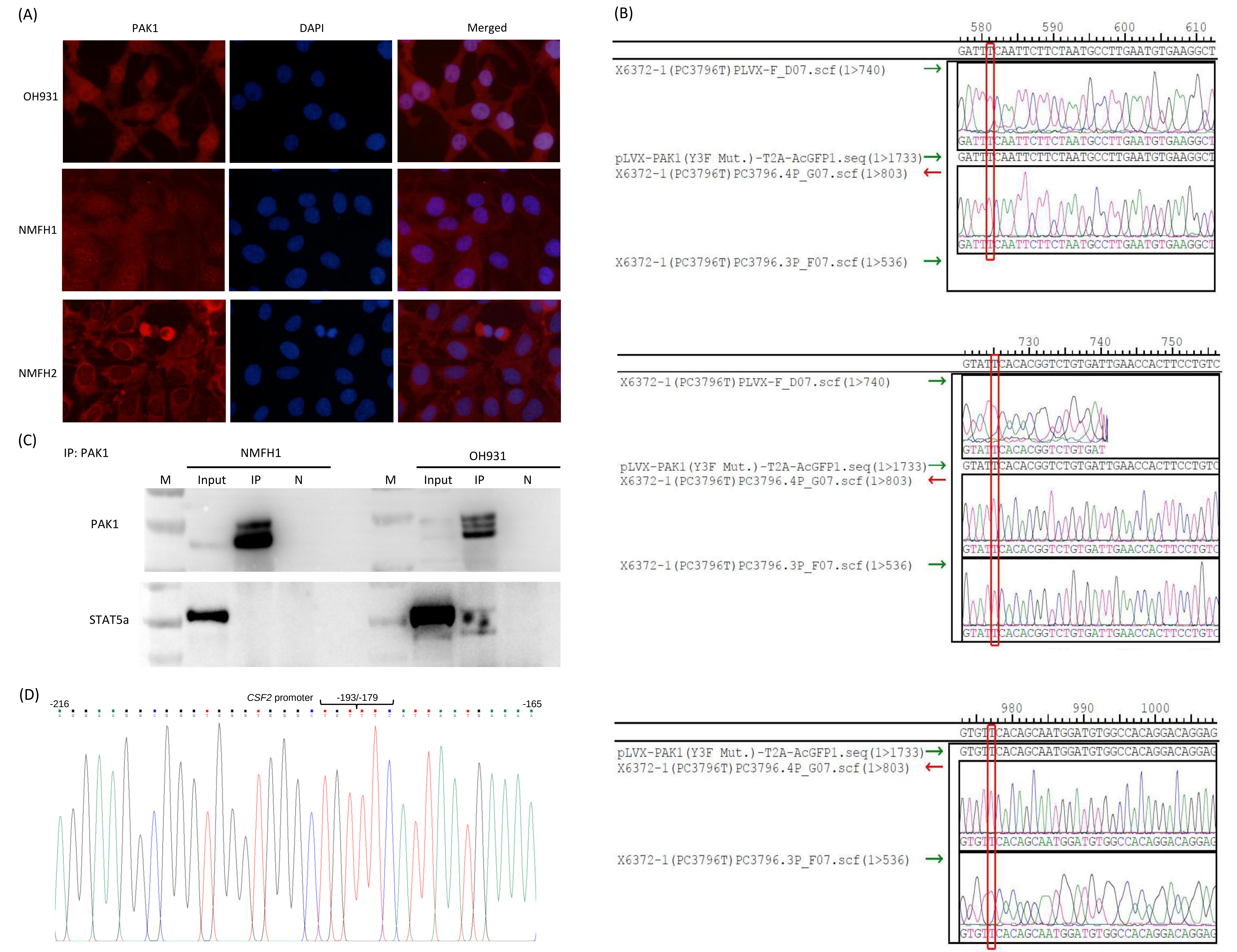
(A)
PAK1
DAPI
Merged
OH931
NMFH1
NMFH2
(B)
580
590
600
610
GATTTCAATTCTTCTAATGCCTTGAATGTGAAGGCT
X6372-1(PC3796T)PLVX-F_D07.scf(1>740)
pLVX-PAK1(Y3F Mut.)-T2A-AcGFP1.seq(1>1733)
X6372-1(PC3796T)PC3796.4P_G07.scf(1>803)
X6372-1(PC3796T)PC3796.3P_F07.scf(1>536)
730
740
750
GTATTCACACGGTCTGTGATTGAACCACTTCCTGTC
980
990
1000
GTGTTCACAGCAATGGATGTGGCCACAGGACAGGAG
(C)
IP: PAK1
NMFH1
OH931
M
Input
IP
N
PAK1
STAT5a
(D)
-216
CSF2 promoter
-193/-179
-165

Figure-S7

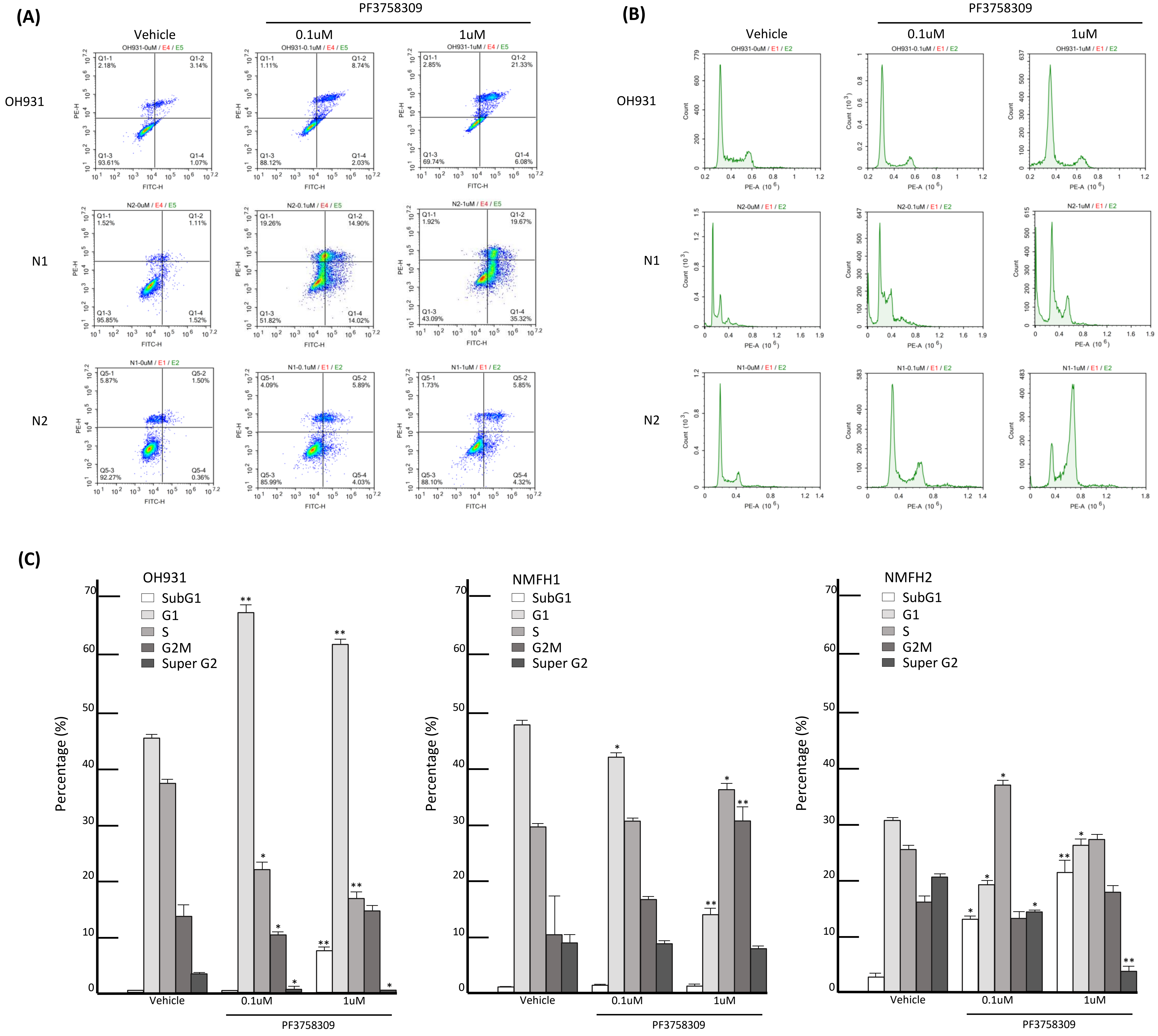
(A)
PF3758309
Vehicle
0.1uM
1uM
OH931
N1
N2
FITC-H
PE-H
(B)
PF3758309
Vehicle
0.1uM
1uM
OH931
N1
N2
PE-A (10 6)
Count
(C)
OH931
NMFH1
NMFH2
SubG1
G1
S
G2M
Super G2
Percentage (%)
Vehicle
0.1uM
1uM
PF3758309

Figure-S8

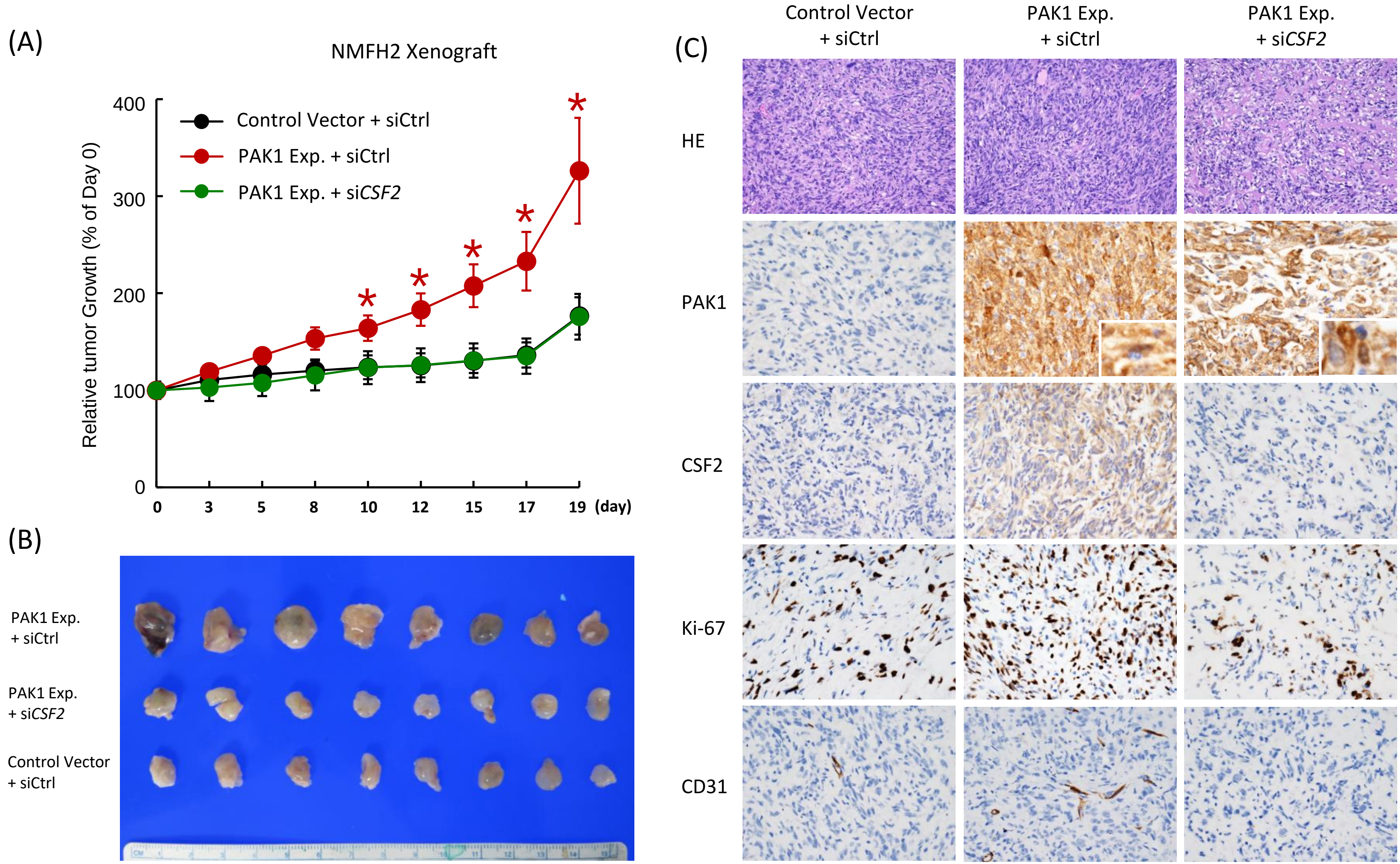
(A)
NMFH2 Xenograft
Relative tumor Growth (% of Day 0)
400
300
200
100
0
Control Vector + siCtrl
PAK1 Exp. + siCtrl
PAK1 Exp. + siCSF2
0
3
5
8
10
12
15
17
19
(day)
(B)
PAK1 Exp. + siCtrl
PAK1 Exp. + siCSF2
Control Vector + siCtrl
(C)
Control Vector + siCtrl
PAK1 Exp. + siCtrl
PAK1 Exp. + siCSF2
HE
PAK1
CSF2
Ki-67
CD31

SUPPORTING INFORMATION FOR ONLINE PUBLICATION ONLY

Supplementary figure legends

Figure-S1. Functional validation of the classic pro-metastatic and pro-proliferative attributes of PAK1 in myxofibrosarcoma cell lines. (A) Compared with those of dermal fibroblasts (CCD966), three myxofibrosarcoma cell lines (NMFH1, NMFH2, OH931) were assessed for the endogenous expression levels of *PAK1* mRNA and protein by quantitative RT-PCR (*upper*) and western blot (*lower*) assays, respectively. **(B)** OH931 *(left)* and NMFH1 *(right)* cell lines were each stably transduced with two different short hairpin RNAs against PAK1 (*shPAK1#1* and *shPAK1#2*), in parallel with their corresponding *shLacZ* controls. The successful genetic ablation of PAK1 expression at the mRNA and protein levels was validated by quantitative RT-PCR (*upper*) and western blots (*lower*), respectively. **(C, D)** In the assays using transwell chambers without (C: cell migration) or with (D: cell invasion) precoated extracellular matrix, the *shLacZ*-transduced OH931 *(left)* and NMFH1 cells (*right*) consistently exhibited significantly higher counts of tumor cells that entered the lower chambers, as compared with their *shPAK1#1*- and *shPAK1#2*-transduced counterparts. **(E)** In the BrdU assay, stable transduction of *shPAK1* with either clone in OH931 (*left*) and NMFH1 *(right)* cells resulted in significantly reduced growth rates, as compared with the *shLacZ* controls from 24 h onward, as plotted in the growth curves. All in vitro assays were performed in triplicate and presented as the mean ± SD. *, P<0.05

Figure-S2. None of PAK2, PAK3, and PAK4 could consistently promote the pro-metastatic and pro-proliferative capabilities in myxofibrosarcoma cell lines. (A) With the expression of control dermal fibroblasts (CCD966) set as 1, the endogenous mRNA and protein levels of PAK2, PAK3, and PAK4 were determined by quantitative RT-PCR (*upper*) and western blot (*lower*) assays, respectively, in three myxofibrosarcoma cell lines (NMFH2, NMFH1, OH931). The mRNA and protein levels of PAK2, PAK3, and PAK4 were consistently higher in NMFH1 and OH931 cell lines, in comparison to NMFH2 cells with generally low abundance of these three PAK kinases. **(B)** Compared with their corresponding *shLacZ* controls, quantitative RT-PCR (*upper*) and western blots (*lower*) confirmed the success of stable transduction of two different short hairpin RNAs each to knock down the expression

of *PAK2* (*shPAK2#1, shPAK2#2*), *PAK3* (*shPAK3#1, shPAK3#2*) or *PAK4* (*shPAK4#1, shPAK4#2*) in OH931 *(left)* and NMFH1 *(right)* cell lines, as evidenced by the decreased levels of mRNA and protein. **(C, D)** As plotted in the histograms and compared with *shLacZ* controls, the migratory capacity (C) was only consistently abolished by either one of two *shPAK4* clones, but no so by *shPAK2* or *shPAK3*, in both OH931 and NMFH1 cells. However, there was no such consistent anti-invasive effect of *shPAK4* in both cell models as observed using transwell chambers precoated with extracellular matrix (D). **(E)** In the BrdU assay, stable transduction of *shPAK3* or *shPAK4* with either of two clones each started exhibiting significantly slower growth rates in NMFH1 cells alone *(right)*, but not in OH931 cells *(left)*, at 72h post-transduction, as compared with the *shLacZ* controls and plotted in the growth curves. All in vitro assays were performed in triplicate and presented as the mean ± SD. *, $P<0.05$.

Figure-S3. PAK2, PAK3, and PAK4 imposed no significant effect on the angiogenesis of myxofibrosarcoma cell lines. In the HUVEC-based angiogenic assay, the formation of capillary tubes was not significantly altered following exposure to conditioned media collected from OH931 *(upper)* and NMFH1 *(lower)* cell lines stably transduced with either of two clones each of *shPAK2* **(A)**, *shPAK3* **(B)** or *shPAK4* **(C)**, as compared with their *shLacZ*-transduced counterparts and plotted in histograms on the right of each panel.

Figure-S4. Ectopic overexpression and hyperactivation of PAK1 can promote cell proliferation, migration, and invasion of the low PAK1-expressing NMFH2 myxofibrosarcoma cell line. (A) The sequencing chromatogram *(upper)* demonstrated the successful site-directed mutagenesis that created a hyperactivated $PAK1^{T423E}$ mutant from the wild-type PAK1 by replacing ACC with GAA in the corresponding codon. As confirmed by qRT-PCR (*lower left*) and western blot (*lower right*) assays and compared with the empty control, wild-type pCMV-PAK1 and the $PAK1^{T423E}$ mutant vectors were stably transfected, resulting in increased PAK1 mRNA and protein in the NMFH2 cells. **(B)** In the BrdU assay, the stable transfectant of pCMV-$PAK1^{T423E}$ revealed the highest growth rate of the proliferative cells as plotted in the histogram. The increased growth rates became significant from Day 2 and Day 3 onward in the pCMV-$PAK1^{T423E}$ and wild-type pCMV-PAK1 transfectants,

respectively. **(C)** Using the scratching method to measure the narrowed width of the wounds, the pCMV-PAK1^{T423E} transfectant of NMFH2 cells showed the most prominent capacity of cell migration, followed by the wild-type pCMV-PAK1 transfectant, as compared with the empty control. **(D)** In the transwell assay, the capacity of cell invasion was confirmed to be the greatest in the pCMV-PAK1^{T423E} transfectant, followed by the wild-type pCMV-PAK1 counterpart and then by the empty control, as plotted in the histogram. All in vitro assays were performed in triplicate and presented as the mean ± SD. *, P<0.05; **, P<0.01.

Figure-S5. Neutralizing anti-CSF2 treatment had no impact on cell viability, migration, and invasion in myxofibrosarcoma cells. As shown in the corresponding histograms, the cell viability determined in the XTT assay (A) and the capacities of cell migration (B) and invasion (C) in the transwell assay were all not significantly altered by the addition of anti-CSF2 antibody (1 μg/ml) in three cell lines.

Figure-S6. Supplementary findings of PAK1 nuclear expression, PAK1^{Y3F} mutant construction, the inconsistent interaction of nuclear PAK1 with STAT5A, and the deletion of *CSF2* promoter construct with confirmatory sequencing. Confocal immunofluorescence **(A)** showed variable but distinct nuclear expression of PAK1 (*red*) with DAPI nuclear counterstain (*blue*) in three myxofibrosarcoma cell lines. The sequencing chromatograms **(B)** confirmed the successful creation of PAK1^{Y3F} mutant to render three tyrosine residues (153, 201, and 285) non-phosphorylatable by using site-directed mutagenesis to replace tyrosine with phenylalanine. **(C)** As probed by western blots, only the IP eluent of OH931 cells showed STAT5A protein pulled down by anti-PAK1, while NMFH1 cells did not reveal physical binding between STAT5A and PAK1. (D) The Sanger sequencing chromatograph validated the deletion of the STAT5 binding site of CSF2 promoter, spanning from -179 to -193 upstream of the transcription start site.

Figure-S7. Flow cytometric analyses confirmed the induction of apoptosis by PF-3758309 with the cell cycle variably arrested at different phases in myxofibrosarcoma cells. (A) As compared with vehicle controls, annexin V-stained apoptotic/necrotic cells were significantly increased by PF-3758309 both at 0.1 μM

and 1 μM. **(B, C)** The induction of apoptosis by PF-3758309 was also evidenced by the variably increased sub$G_{0\text{-}1}$populations as seen in the representative diagrams of cell cycle kinetics (B) as well as in the summarizing histograms (C), in which PF-3758309, both at 0.1 μM and 1 μM, arrested the progression of cell cycle at the G1 or G1/S phase in OH931 and NMFH2 cells and at G2/M phase in NMFH1 cells. *, P<0.05, **, P<0.01. N1: NMFH1; N2: NMFH2

Figure-S8. Suppression of tumor growth by CSF2 knockdown in PAK1-transfected NMFH2 xenografts.

(A) As per caliper measurement, the average tumor volume of NFMH2 xenografts was significantly larger in the *PAK1/siCtrl* group than in the *empty vector/siCtrl* or *PAK1/siCSF2* group from Day 10 onward (n=8 in each group), with this difference remaining significant until Day 19 post-implantation. **(B)** The *PAK1/siCtrl* xenografts were grossly bigger than the *empty vector/siCtrl* and *PAK1/siCSF2* counterparts on Day 19 at sacrifice. **(C)** Microscopically, the hypercellular pleomorphic cells with scant matrix, heightened CSF2 expression, increased CD31-stained microvascular density, and higher Ki-67 proliferative index observed in the *PAK1/siCtrl* xenografts (*middle column*) were all prominently negated in the *PAK1/siCSF2* counterpart (*right column*), even reduced to the degree seen in the *empty vector/siCtrl* control (*left column*). However, strong cytoplasmic and nuclear (inset) PAK1 reactivity was present in both PAK1-transfected groups, regardless of transduction with *siCSF2* or *siCtrl.*

Supplementary Table-S1 Associations of PAK1 and pPAK1 expression with various clinicopathological parameters

	PAK1 Exp. (n=104)			PAK1 mRNA (n=71)		PAK1 gene dosage (n=71)			Whole-cell p-PAK1^{T423E} LI (n=104)			Nuclear p-PAK1^{T423E} LI. (n=104)			CSF2 LI (n=104)		
	**Low**	**High**	**p-value**	**Fold**	**p-value**	**No Amp.**	**Amp.**	**p-value**	**Low**	**High**	**p-value**	**Low**	**High**	**p-value**	**Low**	**High**	**p-value**
**Sex**[&]			0.191		0.401			0.315			0.078			0.290			0.839
Male	39	21		0.93±0.85		37	5		21	23		44	16		36	24	
Female	23	21		1.09±0.70		23	6		39	21		28	16		28	16	
**Age**[#]	59.85±14.16	61.62±14.27	0.536	r=-0.026	0.830	61.13±13.18	58.55±18.95	0.579	59.03±14.88	62.66±12.98	0.198	14.40±1.70	13.81±2.44	0.756	59.13±14.67	62.88±13.16	0.166
**Location**[&]			0.422		0.315			0.650			0.106			0.846			0.713
Extremity	46	34		0.93±0.69		43	7		43	37		55	25		50	30	
Axial	16	8		1.13±0.97		17	4		17	7		17	7		14	10	
**FNCLCC grade**[&]			**<0.001**		**<0.001***			**0.034***			**<0.001***			**<0.001***			0.402
grade 1	37	8		0.75±0.50		29	3		36	9		40	5		31	14	
grade 2	20	25		0.99±0.72		26	4		18	27		26	19		25	20	
grade 3	5	9		1.91±1.16		5	4		6	8		6	8		8	6	
**AJCC stage**[$]			**0.009***		**0.034***			**0.016***			**0.004***			**0.007***			0.095
Stage 1 and 2	38	15		0.84±0.68		32	2		37	16		42	11		37	16	
Stage 3	17	21		1.29±0.94		20	8		15	23		20	18		20	18	
**Tumor size**[#]	6.17±5.27	7.44±4.47	0.224	r=0.034	0.787	6.78±5.09	8.25±6.01	0.414	6.016±5.20	7.64±4.55	0.116	6.22±4.97	7.78±4.93	0.163	6.529±5.07	6.767±4.80	0.612
**Mitotic rate**[#]	7.50±7.66	15.43±13.48	**<0.001***	r=0.425	**<0.001***	9.00±9.37	17.91±15.57	**0.012***	7.97±8.76	14.43±12.78	**0.003***	8.19±8.83	16.34±13.44	<0.001*	9.89±11.08	12.00±11.06	0.209
**Microvascular density (%)**	11.80±8.31	15.76±8.86	**0.012***	r=0.235	**0.048***	12.86±8.48	22.23±9.42	**0.002***	10.38±6.77	17.53±9.43	**<0.001***	11.02±7.48	18.76±9.02	**<0.001***	10.91±7.30	17.40±9.39	**<0.001***
**PAK1 LI**[#]					**<0.001***	-	-	**0.026***			**<0.001***			**<0.001***			**0.001***
Low Exp.				0.72±0.50		38	3	-	49	13		53	9		46	16	
High Exp.				1.38±0.94		22	8		11	31		19	23		18	24	
**PAK1 mRNA**[#]	XX	XX	XX			0.78±0.52	2.18±0.96	**<0.001***	0.78±0.70	1.28±0.82	**0.006***	0.80±0.63	1.48±0.92	**<0.001**	0.73±0.45	1.34±0.98	**0.002***
**Gene dosage**[&]											**0.034***			**0.007***			**0.034***
No Amp.						-	-	-	37	23		46	14		37	23	
Amp.						-	-	-	3	8		4	7		3	8	
**Whole-cell p-PAK1^{T423E} LI**[&]														**<0.001***			**<0.001**
Low Exp.												59	1		46	14	
High Exp.												13	31		18	26	

**Nuclear p-PAK1^{423} LI**																	**<0.001***
Low Exp.															56	16	
High Exp.															8	24	

LI, labeling index denoting percentage of tumor cells exhibiting cytoplasmic and/or nuclear expression as specified in the method for each immunohistochemical variable

*: Statistically significant, &: Chi square test, #: Mann–Whitney U test; $: 91 cases assessable for staging

Table-S2 Univariate log-rank analyses for prognostic factors in 97 patients with follow-up

Parameters	Category	No. of FU	DSS		MeFS	
			No. of event	*P*-value	No. of event	*P*-value
**Sex**	Male	56	10	0.9145	12	0.2744
	Female	41	6		13	
**Age**	<60 years	34	6	0.8657	8	0.4602
	≧60 years	63	10		17	
**Location**	Extremity	76	11	0.1158	18	0.1774
	Axial	21	5		7	
**Tumor size**	<6 cm	50	6	0.3329	8	**0.0199***
	≧6 cm	42	7		14	
**Mitotic count**	<20/10 HPFs	78	8	**0.0001***	13	**<0.0001***
	≧20/10 HPFs	19	8		12	
**FNCLCC grade**	Grade 1	41	2	**<0.0001***	3	**<0.0001***
	Grade 2	42	7		12	
	Grade 3	14	7		10	
**AJCC stage$**	Stage 1 & 2	47	5	0.1119	8	**0.0181***
	Stage 3	37	8		14	
**PAK1 LI**	Low Exp.	56	6	**0.0474***	9	**0.0036***
	High Exp.	41	10		16	
***PAK1* mRNA#**		61	H.R.=3.170	**<0.0001***	H.R.=2.822	**<0.0001***
***PAK1* gene dosage&**	No Amp.	57	4	**<0.0001***	9	**<0.0001***
	Amp.	11	6		8	
**Whole-cell p-PAK1^{T423} LI**	Low Exp.	54	4	**0.0117***	5	**<0.0001***
	High Exp.	43	12		20	
**Nuclear pPAK1^{T423} LI**	Low Exp.	66	3	**<0.0001***	6	**<0.0001***
	High Exp.	31	13		19	
**CSF2 LI**	Low Exp.	58	3	**0.0005***	6	**0.0001***
	High Exp.	39	13		19	

DSS, disease-specific survival; MeFS, metastasis-free survival;

*, Statistically significant p values; $, Case number = 84; &, Case number = 68; #, case number=61

Table-S3 Multivariate survival analysis

Parameters	Category	DSS			MeFS		
		H.R.	95% C.I	*P*-value	H.R.	95% C.I.	*P*-value
**FNCLCC Grade**	Grade 1	**1**	**-**	**<0.001***	**1**	**-**	**<0.001***
	Grade 2	**1.657**	**0.328-8.371**		**1.667**	**0.357-7.778**	
	Grade 3	**22.327**	**3.655-148.863**		**30.664**	**4.693-200.368**	
**Whole-cell p-PAK1^{T423} LI**	Low Exp.	1	-	0.104	1	-	**0.018***
	High Exp.	3.116	0.793-12.248		3.992	1.265-12.598	
**CSF2 LI**	Low Exp.	1	-	**0.014***	1	-	0.110
	High Exp.	6.291	1.446-27.368		2.640	0.802-8.694	
**AJCC stage**	Stage 1 & 2	-	-	-	1	-	0.741
	Stage 3	-	-	-	0.850	0.323-2.237	

Supplementary methods

Method-S1 Quantigene Branched-chain DNA in situ hybridization (bDISH) assay

A sandwich nucleic acid hybridization assay was applied to quantitate the mRNA abundance of *PAK1* and housekeeping genes in tissue homogenates of formalin-fixed specimens. Briefly, specific probes targeting *PAK1* transcript were customized by QuantiGene Multiplex 2.0 assay system (Affymetrix/Panomics). Oligonucleotides of the probe set were mixed with the lysed formalin-fixed tissues, and the mixture was added to a 96-well plate coated with capture probe oligonucleotides. Target RNA was captured and incubated overnight at 55 °C with removal of unbound material using 300 µl wash buffer for 3 runs, followed by hybridization of DNA amplifier molecules and three additional washes after incubation every time. The dioxetane alkaline phosphatase substrate Lumiphos Plus was added to the reaction wells for detection by Luminex 100 microplate luminometer (Luminex). The detected readout of *PAK1* mRNA abundance was further normalized by the expression level of reference *GAPDH* transcript.

Method-S2 Cell culture, RNA interference, and transfection

With 10^5 cells each seeded in a 24-well plate and incubated with lipofectamine 2000 (Invitrogene), PAK1-underexpressing NMFH2 cell lines were stably transfected with 1.5µg empty pCMV6, wild-type pCMV6-PAK1, or hyperactive mutant pCMV6-PAK1^{T423E} mutant vector tagged with DDK-Myc and selected with neomycin for stable clones. The former two were purchased from Addgene and validated by sequencing. The mutation forms of PAK1 including pCMV6-PAK1^{T423E} and pCMV6-PAK1^{Y3F} mutants were created by oligonucleotide synthesis and validated by direct sequencing (Topgen Biotechnology Co., Ltd). The transfected cells were analyzed for the expression of exogenous PAK1 and tagged proteins by western blotting.

In comparison to pLKO.1-*shLacZ* (TRCN0000072223: 5’-TGTTCGCATTAT CCGAACCAT-3’) as the non-targeting control, pLKO.1-*shPAK1* (TRCN0000002224, 5’- CCAAGAAAGAGCTGATTATTA -3’; TRCN0000002226, 5’-GCGATCCTAAGAAGAAATATA-3’), pLKO.1-*shPAK2* (TRCN0000002115: 5’-CTCTAGGAACCAAAGTGATTT-3’; TRCN0000194671, 5’-CGGGATTTCTTAAATCGATGT-3’), pLKO.1-*shPAK3* (TRCN0000003243: 5’-CAACCCAAGAAGGAATTAATT-3’; TRCN0000195142, 5’-CTTGGAATTATGGCAATTGAA-3’), and pLKO.1-*shPAK4* (TRCN0000010198:

5’-CGAGAATGTGGTGGAGATGTA-3’; TRCN0000010201, 5’-CTGCTGGACGAGTTTGAGAAC-3’) lentiviral vectors were obtained from Taiwan National RNAi Core Facility and individually transduced into OH931 and NMFH1 cells. Viruses were prepared in HEK293 cells co-transfected with packing plasmids and the above short hairpin RNAs using Lipofectamine 2000. For viral infection, 3×10^6 cells were infected with lentiviral supernatant containing polybrene (8 μg/ml), followed by selection with puromycin for stable clones of lentivirus-transduced cells.

To assess the angiogenic effect of CSF2 in the context of PAK1, pre-designed si*CSF2* (Ambion, Assay ID: S3597) or negative control siRNA (Ambion) was transiently transduced into myxofibrosarcoma cells stably transfected with wild-type PAK1 or empty vector. To analyze the dependency of PAK1-dictated CSF2 transactivation on STAT5, two pre-designed short interfering RNAs targeting STAT5 (si*STAT5b*, Ambion, Assay ID: s13538) or their negative control siRNA (si*Ctrl*, Ambion) were transiently transduced using Lipofectamine® RNAiMAX reagent (Invitrogen) into the transfectants of all three myxofibrosarcoma cell lines bearing wild-type pCMV6-PAK1 and the luciferase-tagged pGL4-phCSF2 promoter construct with or without deleting the STAT5B binding site (also see method-S6 below).

Method-S3 qRT-PCR for *PAK1/2/3/4, STAT5b* and *CSF2* transcripts

Extracted RNAs were reverse-transcribed with SuperScript™ III first-strand synthesis kit (Invitrogen). By using pre-designed TaqMan assay reagents (Applied Biosystems), ABI StepOnePlus™ Real-Time PCR System was employed to quantitate mRNA abundance in variously genetically manipulated cell models and corresponding controls for transcripts of *PAK1* (Hs00945621_m1), *PAK2* (Hs01127126_m1), *PAK3* (Hs01040810_m1), *PAK4* (Hs01100061_m1), *STAT5b* (Hs00560026_m1), and *CSF2* (Hs00929873_m1) (Thermo Fisher, MA). The data were normalized to *POLR2A* housekeeping transcript (Hs01108284_g1). The expression of target transcript was given by $2^{-\Delta\Delta C_p}$, where $\Delta\Delta C_T = \Delta C_{T\ (target)} - \Delta C_{T\ (reference)}$ and ΔC_T represented the C_T of target transcript subtracted from the C_T of *POLR2A*. Only samples with C_T value <32 for *POLR2A* were considered acceptable in quality for analysis.

Method-S4 Human angiogenesis antibody array

Proteome Profiler™ Human Angiogenesis Array Kit (R&D, ARY007) was applied to search for differentially expressed angiogenesis-regulated molecules between *shPAK1* and *shLacZ* transductions for both PAK1-overexpreassing OH931 and

NMFH-1 cell lines. In brief, the angiogenesis antibody membrane, spotted with 55 immobilized angiogenesis-associated antibodies in duplicate, was placed onto the 4-well multi-dish, immersed in 2 ml of array blocking buffer, incubated for 1 hour, followed by removal of the blocking buffer and washed twice. In a single sample , 1.5 ml of lysates from 10^6 cells, rinsed with PBS and lysed with provided buffer, was placed on the membrane, which was then incubated overnight at 4 °C, washed with 20 mL of 1X array wash buffer, continued for incubation at room temperature for 5 minutes, and added with 2 ml of streptavidin-HRP solution for 30 min incubation. The membrane was stringently washed trice before treatment with enhanced chemiluminescence (Thermo Scientific). The ChemiDoc™ Touch Imaging System (Bio-Rad) was employed to take images of the array membranes and the Image Lab Software 5.2.1 (Bio-Rad) to quantify the mean spot pixel density of chemiluminescence for analyzing the differentially expressed proteins.

Method-S5 Western blots

Three myxofibrosarcoma cell lines were analyzed by western blots to evaluate their endogenous expression of total PAK1 and phospho-PAK1, the efficiency of silencing PAK1 in NMFH-1 and OH931 cells and transfecting wild-type pCMV6-PAK1 and mutant pCMV6-PAK1^{T423E} in NMFH2 cells, the physical interaction between PAK1 and STAT5 following co-immunoprecipitation assay, and the effect of pharmacological inhibition with PF3758309. Whole cell lysates were minced in RIPA lysis buffer with phosphatase and protease inhibitors, homogenized, and quantified by Bio-Rad Assay Kit. Equal amounts of protein extracts were separated on SDS-10% PAGE and transferred to nitrocellulose membranes for the following purposes: The membranes were blocked with 5% skimmed milk and probed overnight using primary rabbit monoclonal antibodies against total PAK1 (Cell signaling, 2602, 1:2000), phospho-PAK1^{T423} (Cell signaling, 2601, 1:2000), STAT5A (Santa Cruz, sc-166479, 1: 2000), STAT5B (Santa Cruz, sc-1656, 1:2000), CSF2 (Abcam, ab300495, 1:1000), caspases-3 (Abcam, ab32042, 1:500) and cleaved caspases-3 (Abcam, ab32042, 1:500). Following incubation with the secondary antibody, the probed proteins were visualized by enhanced chemiluminescence reagents under densitometry, with GAPDH protein (Chemicon) serving as a loading control.

Method-S6 Luciferase reporter assay for measuring *CSF2* promoter activity

The pGL4-ph*CSF2* promoter construct (RIKEN, Japan) was employed in the

reporter assay, where the pGL4 luciferase reporter vector was inserted with a human *CSF2* promoter sequence (1487bp, spanning -1383 to +35 relative to the *CSF2* transcription start site), in which a critical STAT5 binding site between -193 and -179 was predicted by using TRANScription FACtor database (TRANSFAC®, geneXplain). In addition, a deletion variant of the pGL4-ph*CSF2* promoter construct lacking the STAT5 binding site (pGL4-ph*CSF2*-Del-193/-179) was produced by site-direct mutagenesis using the following primers: Forward: 5'-AGCCTCAGGCCCATTCAGAC-3'; Reverse: 5'-TGAGCCTTTTCCCTAGGTGG-3'. According to the manufacturer's instructions, the pGL4-ph*CSF2* or the STAT5 binding-deleted construct was co-transfected with a Renilla vector as a baseline control into myxofibrosarcoma cell lines with various genetic manipulations (*shPAK1*, pCMV6-PAK1, pCMV6-PAK1^{T423E}, pCMV6-PAK1^{Y3F}, siSTAT5 and their corresponding controls) using PolyJet™ transfection reagent (SignaGen Laboratories, Gaithersburg, MD). At 48 hour post-transfection, cells were subjected to ONE-Glo™ Luciferase Reporter Assay (Promega), following the manufacturer's instructions. Luciferase activity of *CSF2* promoter were normalized to the readout of Renilla activity.

Method-S7 Chromatin immuneprecipitation (qChIP) for *CSF2* promoter

The Magna ChIP A/G chromatin Immunoprecipitation Kit (Millipore, 17–10085) was coupled with qPCR to perform qChIP assay on various stable PAK1-manupilated or PF-3758309-treated myxofibrosarcoma cells fixed with 1% formaldehyde for 15 min. The cross-linked chromatin was sheared to an average size of 500-1000 bp, followed by precipitation of DNA fragments at 4°C overnight with Dynabeads Protein A and antibodies against total PAK1 (Cell signaling, 2602, 1:200) and STAT5B (Santa Cruz, sc-1656, 1:200) or control rabbit immunoglobulin G (Abcam, ab171870, 1:100) at 4°C overnight. After reversing the cross-linking, the precipitated chromatin DNA is quantitated by qPCR targeting the *CSF2* promoter using cyber green-based chemistry. The primer sequences used for qPCR were as follows: Forward: 5'-CGGGTGGGTGGGCTGTTTCATTAATGAAAACCC-3'; Reverse: 5'-GGGTTTTCATTAATGAAACAGCCCACCCACCCG-3'.

Method-S8 Co-immunoprecipitation (co-IP)

Co-IP assays were performed using Dynabeads® Protein G to immunoprecipitate the lysates from OH931, NMFH1, and NMFH2 cell lines. In brief, anti-PAK1

(Abcam, ab223849, 1:1000) was used as the bait coupled to the beads using DMP (Sigma) as cross-linkers following manufacturer's instructions. Lysates were cleared by centrifugation, quantified, and then incubated with the anti-PAK1-conjugated beads for 1 hour, washed trice with 10 mM Tris-HCl/50 mM KCl and equally split into 6 new tubes. For elution, the beads were heated for 10 minutes at 70°C in 20 μl of NuPage SDS-containing loading buffer and for 10 more minutes at 70°C in LDS buffer. Western blots with the following dilution folds for anti-STAT5B (Santa Cruz, sc-1656, 1:100), anti-STAT5A (Santa Cruz, sc-166479, 1: 200), and anti-phospho-tyrosine (Cell signaling, P-Tyr-1000, 1:2000) were then conducted on the eluent to assess the interacting proteins bound to the beads using another antibody other than the bait.

Method-S9 Confocal immunocytochemistry

To confirm the dictation of tyrosyl phosphorylation in PAK1 nuclear entry, confocal immunoflorescent microscopy (FV10i and FV3000, Olympus) was used to visualize the redistribution of PAK1 expression between subcellular nuclear and cytosolic compartments in the pCMV6-PAK1^{Y3F}-transfected NMFH2 cells versus the empty pCMV6 control, with anti-PAK1 and anti-Myc being labeled with Texas red and GFP, respectively. To ascertain the co-localization of PAK1 with STAT5B, the subcellular distribution of endogenous PAK1 and STAT5B in all three parent myxofibrosarcoma cell lines, with anti-PAK1 and STAT5B being labeled with Texas red and GFP, respectively. Cells grown on glass coverslips were methanol-fixed, incubated with primary antibodies and Alexa Fluorescent dyes-conjugated secondary antibodies (Molecular Probes), and nuclear-counterstained with DAPI. Sequential laser excitation was applied to minimize fluorescent emission, with images reconstructed by *Z* stacks of serial sections at the same cellular level and magnification.

Method-S10 Bromodeoxyuridine (BrdU) assay to assess DNA synthesis

DNA synthesis was assessed using an enzyme-linked immunosorbent assay-based and colorimetric BrdU assay (Roche Diagnostics). Myxofibrosarcoma cells with various genetic manipulations, including pCMV-PAK1 vs. pCMV empty vector, *shRSF1* vs. *shLacZ* control or *siIL1B* vs. *siCtrl,* were plated into a 96-well plate at density of 3000 cells per well. DNA synthesis was evaluated at 24, 48, and 72 h. After incubation with BrdU for 3 hours at 37°C under 5% CO_2, the labeling medium was removed, followed by fixation and final incubation with anti-BrdU-POD solution. The

absorbance of the samples was measured using an ELISA reader (Promega) at 450 nm, with the absorbance at 690 nm as reference.

Method-S11 Wound-healing migration

An artificial "wound" was scratched using a 200 ul pipette tip on confluent cell monolayers in 6-well plates. Photographs were taken at 0, 4, 8, 12, and 24 hrs. Quantitative analysis of the wound closure was calculated by measuring the percentage of healed area relative to the initial wound on Day 0.

Method-S12 invasion assays

Transwell cell invasion assays were determined using gels consisting of 24-wells with pre-coated extracellular matrix (Millipore). In brief, each insert was added with 0.3 ml serum-free medium, replaced with 0.3 ml of serum-free suspension of 10^6 cells in the upper chamber, and incubated for 24 hr to let cells move across the lower chamber containing 10% FBS. After removing cells in the upper chamber, cells invading through the inserts were stained with the provided dye, dissolved in extraction buffer, and transferred to 96-well plates for colorimetric reading at 560 nm.

Method-S13 cell cycle kinetic analysis

In OH931, NMFH1, and NMFH2 myxofibrosarcoma cell models, stable pools of lysates of PF3758309-treated cells at indicated doses versus the corresponding vehicle control were pelleted and fixed overnight in 75% cold ethanol at −20°C. Cells were washed twice in cold PBS containing 10 mg/ml of DNase-free RNase. Afterwards, these cells were labeled with propidium iodide (PI) at a concentration of 0.05 mg/ml and analyzed by FACScan flow cytometer (BD Biosciences) with WinMDI2.9 software to determine the percentage of cells in each phase of the cell cycle. In all experiments, at least more than 10^4 cells were sorted after gating out the fixation artifacts and cell debris.

Method-S14 Evaluation of apoptosis by annexin V/propodium iodine staining

For apoptosis analysis, 10^5 cells from each of 3 myxofibrosarcoma cells, treated with indicated doses or the corresponding vehicle control, were plated for 24 h and incubated with Annexin V-FITC kit (Bender MedSystems, CA) containing propodium iodine for 15 min. The cell percentages at the stages of early apoptosis and late apoptosis, and necrosis were calculated from three independent experiments.

Method-S15: HUVEC-based angiogenic assay

HUVEC assays were applied to analyze the effects of PAK1, PAK1^{T423E}, PAK2/3/4, CSF2, CSF2-neutralizing antibody, or PF3758309 treatment on

myxofibrosarcoma cells-induced endothelial tube formation. The conditioned media of these genetically or pharmacologically manipulated cells, as well as their corresponding controls, were harvested by serum-free starvation for 96 h. HUVEC cells were then plated at a density of 2 x 10^4 cells/well and incubated with various conditioned media. After 6 h of treatment, the capillary-like tube formation was assessed by measuring the length of the capillary mesh under an inverted photomicroscope.
